# International comparison of pharmaceutical industry payment disclosures in the UK and Japan: implications for self-regulation, public regulation, and transparency

**DOI:** 10.1186/s12992-022-00902-9

**Published:** 2023-03-03

**Authors:** Piotr Ozieranski, Hiroaki Saito, Emily Rickard, Shai Mulinari, Akihiko Ozaki

**Affiliations:** 1 Department of Social and Policy Sciences, University of Bath, Claverton Down, Bath, BA2 7AY UK; 2 Department of Gastroenterology, Sendai Kousei Hospital, Sendai, Miyagi Japan; 3 Department of Sociology, Lund University, Lund, Sweden; 4 Department of Breast Surgery, Jyoban Hospital of Tokiwa Foundation, Iwaki, Fukushima Japan

**Keywords:** Comparative case study, Transparency, Financial conflicts of interest, Payment disclosure, Research and development, Self-regulation, Pharmaceutical industry

## Abstract

**Background:**

Self-regulation of payment disclosure by pharmaceutical industry trade groups is a major global approach to increasing transparency of financial relationships between drug companies and healthcare professionals and organisations. Nevertheless, little is known about the relative strengths and weaknesses of self-regulation across countries, especially beyond Europe. To address this gap in research and stimulate international policy learning, we compare the UK and Japan, the likely strongest cases of self-regulation of payment disclosure in Europe and Asia, across three dimensions of transparency: disclosure rules, practices, and data.

**Results:**

The UK and Japanese self-regulation of payment disclosure had shared as well unique strengths and weaknesses. The UK and Japanese pharmaceutical industry trade groups declared transparency as the primary goal of payment disclosure, without, however, explaining the link between the two. The rules of payment disclosure in each country provided more insight into some payments but not others. Both trade groups did not reveal the recipients of certain payments by default, and the UK trade group also made the disclosure of some payments conditional on recipient consent. Drug company disclosure practices were more transparent in the UK, allowing for greater availability and accessibility of payment data and insight into underreporting or misreporting of payments by companies. Nevertheless, the share of payments made to named recipients was three times higher in Japan than in the UK, indicating higher transparency of disclosure data.

**Conclusions:**

The UK and Japan performed differently across the three dimensions of transparency, suggesting that any comprehensive analysis of self-regulation of payment disclosure must triangulate analysis of disclosure rules, practices, and data. We found limited evidence to support key claims regarding the strengths of self-regulation, while often finding it inferior to public regulation of payment disclosure. We suggest how the self-regulation of payment disclosure in each country can be enhanced and, in the long run, replaced by public regulation to strengthen the industry’s accountability to the public.

## Background

Collaborations between pharmaceutical companies and the healthcare sector facilitate drug development and commercialisation, disease awareness, and disseminating information on medicine use [1–5]. They have become ever more pronounced as new specialist drugs, including for genetic diseases and some cancers, proliferated, requiring advice from medical experts and researchers throughout the product life cycle [6–10]. Similarly, decreasing industry investment in in-house drug development has emphasised academic partnerships as sources of innovation [11–14].

However, these collaborations may generate financial conflicts of interest (FCOIs) biasing healthcare practice, research, education, and policy [15–17]. The main global response to these concerns is disclosure of drug company payments – including for consultancies, sponsorships, and grants – to healthcare professionals (HCPs) and organisations (HCOs) [18–20]. Payment disclosure involves different “transparency provisions”: (1) self-regulation, primarily voluntary codes of conduct developed and enforced by industry trade groups; (2) public regulation, typically sunshine legislation mandating drug companies to disclose certain payments to some recipient categories; or (3) their combination [18, 21–25].

With few exceptions [26], research to-date has utilised single-case studies to examine the strengths and weaknesses of public regulation in the US [23, 27, 28] and France [29]; and self-regulation in the UK [30–32], Germany [10, 33], Ireland [34], Australia [18, 35–37], and Japan [38–44]. There have been fewer direct comparisons of countries with public regulation and self-regulation [18, 21, 22, 24, 45, 46]. Compared with public regulation, self-regulation of payment disclosure is described by its proponents as less burdensome, more flexible and responsive to the needs of a globalised industry [10, 25, 45, 47–49]. Strengths of public regulation include mandatory disclosures and more healthcare industries and payments covered by the requirements [18, 22, 45]. Crucially, both are critiqued for low disclosure accessibility, quality, and user-friendliness [21, 24].

Against this background, except for Europe [24, 26, 46], the relative strengths and weaknesses of self-regulation of payment disclosure across countries are poorly understood. Nevertheless, the variability of European cases is low as nearly all national trade groups follow – but rarely exceed – the minimum requirements from the Code of Practice of the European Federation of Pharmaceutical Industries and Associations (EFPIA) [21, 24, 50, 51]. What also underscores the need for comparative analysis of self-regulation of payment disclosure is its global prevalence [36]. Indeed, it is the default approach in countries without public regulation [51] and only two of the largest pharmaceutical markets – the US and France – have comprehensive sunshine legislation, while other known examples have limited scope or exist alongside self-regulation [18, 21, 24]. Self-regulation is also an important policy tool, e.g. in benchmarking [18], with country rankings of disclosure statistics and code compliance becoming an argument against the introduction of sunshine legislation [47, 52, 53].

We seek to compare the strengths and weaknesses of self-regulation of payment disclosure using two crucial case studies: the UK and Japan. While each country has been analysed separately, the focus has been on select payments to HCPs and HCOs [30, 31, 38, 39, 41, 42, 54]. Disparate research designs have further constrained possibilities for systematic analysis of the uniqueness and commonalities relative to other cases, reducing generalisable international policy learning [30, 31, 38]. In addressing these limitations, we use a typology of dimensions of transparency developed in internationally comparative research on payments to patient organisations, an area of industry self-regulation separate from the scope of this article [55]. We examine three such dimensions: (1) the scope of payment disclosure *rules* established by the industry trade groups; (2) how payments are disclosed *in practice*; and (3) the insight into industry-healthcare sector financial relationships generated by the payment *data*.

Japan and the UK have the third and seventh largest pharmaceutical markets worldwide [56], with extensive drug manufacturing and research and development (R&D) capacity [57, 58]. They are likely the best cases of self-regulation of payment disclosure in Europe and Asia, and potentially globally, having been characterised as more comprehensive than another established example of self-regulation, Australia [36, 38].

Since 2015, the Code of Practice of the Association of the British Pharmaceutical Industry (henceforth ABPI Code) has included payments and recipients exceeding EFPIA’s minimum requirements [24, 31, 59]. Despite important shortcomings [30, 31], the transparency of disclosures by companies following the ABPI Code is the highest within the EFPIA ecosystem, especially following its major revision in 2021, which introduced the disclosure of additional payment and recipient categories [21, 24]. Further, while only around two-thirds of individuals receiving payments consent to disclosure [60], this reflects the pattern observed in most European countries where industry interprets payment data as falling under the European privacy laws [26, 30, 47, 52, 53].

As far as we know, since 2011 the Japan Pharmaceutical Manufacturers Association (JPMA) has developed Asia’s most comprehensive standards on interactions with the healthcare sector. Specifically, the “Transparency Guidelines for the Relation between Corporate Activities and Medical Institutions” (henceforth JPMA Transparency Guidelines) [61], focus on payment disclosure, while the “JPMA Code of Practice” (henceforth JPMA Code) [62] covers promotion.

### Context of self-regulation of payment disclosure in the UK and Japan

The pharmaceutical industry globally faces similar political and regulatory pressures to enhance transparency [18, 21, 25, 36, 49], potentially resulting in similar approaches to disclosure. In the UK, key manifestations of these pressures have included a parliamentary investigation into the industry’s influence on healthcare provision [63] and a medical product safety review [64, 65]. Controversy has also been caused by the Japanese company Astellas’ illicit marketing, resulting in its temporary suspension from the ABPI [48, 66]. In Japan, several well-publicised scandals have involved kickbacks to increase prescription [41] and data fabrication in clinical trials [44, 67–71]. The ensuing criminal investigations have prompted policy discussions on transparency, leading to the development of the new Clinical Trials Act [44, 72, 73].

As members of the International Federation of Pharmaceutical Manufacturers and Associations (IFPMA) [74, 75], the ABPI and JPMA subscribe to its Code of Practice [62, 74] and transparency guidelines on fees for services [76], sponsorship of events and meetings [77], and continuing medical education [78]. They also share company members, including the UK-headquartered GSK and AstraZeneca and Japan-headquartered Takeda and Astellas [79–82]. Like other multinationals, their global codes of conduct [49, 51] make similar commitments to transparency [83–86] and payment disclosure [85, 86]. The ABPI and JPMA collaborate [87, 88], including via EFPIA Japan [89], an organisation representing European company interests which contributed to the development of the JPMA Transparency Guidelines [90].

Although the “broad parameters” of payment disclosure are shared internationally, detailed expectations vary across jurisdictions [18, 25]. Indeed, IFPMA expects its member associations to implement codes reflecting national circumstances [49]. Therefore, the ABPI implements EFPIA’s standards [74] that exceed IFPMA’s requirements regarding payment disclosure [51]. In comparison, the JPMA responds, among others, to the expectations of domestic fair trade and marketing organisations [91].

Payment disclosure is also affected by the trade groups’ varying governance structures. As part of EFPIA’s compliance monitoring, the ABPI publishes yearly evaluations of company compliance with its Code [47, 52, 53]. Complaints regarding alleged breaches of the ABPI Code are considered by the Prescription Medicines Code of Practice Authority (PMCPA), a quasi-autonomous body within the ABPI [92, 93]. The PMCPA publishes reports of all complaints and levies “administrative charges” on breaching companies, and in instances of particularly problematic company behaviour, it can rule a breach of §2 of the Code (promotion that “brings discredit to, and reduction of confidence”) and can issue a public reprimand [92]. Contrastingly, because the Transparency Guidelines are voluntary for its member companies, the JPMA does not monitor company adherence or sanction non-compliance [38, 94]. However, the JPMA’s executive board investigates complaints regarding alleged breaches of the JMPA Code, which is distinct to the Transparency Guidelines, and decides on penalties, but does not publish their details systematically [94].

Differences between each country’s “medical economy” are also significant [91, 95, 96]. Although both have publicly funded healthcare offering universal coverage to residents [57], Japan combines physician-owned hospitals and clinics with a sizeable sector of hospitals owned by public authorities and a few not-for-profit hospitals run by charities or foundations [58, 91]. Contrastingly, in the UK, healthcare is predominantly delivered by practice-owning physicians, who are patients’ first point of contact, and salaried staff in publicly-owned National Health Service (NHS) hospitals [97].

To some extent, these health-system level differences entail different patterns of the industry-healthcare sector interactions. Like in other major pharmaceutical markets [91], in the UK and Japan, industry marketing targets physicians, including medical Key Opinion Leaders (KOLs) [63, 94, 98, 99] and hospitals [41, 100]. The JPMA has also sought to regulate financial ties pertinent to Japan, such as extensive funding of physicians’ and university research [91]. Similarly, while attempts at establishing reciprocity and loyalty with physicians have been reported internationally [101–103], their role might be more pronounced in Japan, given the cultural importance of informal gifts, associated with evidence of bribes and kickbacks in medical procurement, prescription, and clinical trials [91].

Lastly, self-regulation belongs to wider mechanisms governing interactions between pharmaceutical companies and the healthcare sector [10, 51, 104]. In Japan, FCOIs are regulated chiefly via the Physicians’ Act, the Medical Service Act, and the Health Insurance Act, which criminalise bribes, kickbacks and gifts to publicly employed physicians [91]. In addition, the Fair Competition Code [105] determines how recipients can spend company payments [91, 105], while the Clinical Trials Act mandates financial disclosure by researchers and research institutions [44, 73]. Although the Clinical Trials Act does not mandate disclosure from drug companies, the study types from the Clinical Trials Act were voluntarily adopted in the JPMA Transparency Guidelines in 2018 in relation to the reporting of R&D payments and expenditure (no changes were introduced to the reporting of non-R&D payments). Nevertheless, FCOI regulation by professional organisations [91] and hospitals [106] has been historically weak, with reports of senior medics seeking to maintain close industry ties [42, 94].

In the UK, the overarching approach to undue influence is covered by the Bribery Act, making bribery a corporate offence [45, 93]. The NHS has voluntary guidance on FCOI disclosure, which is characterised by important loopholes [107, 108]. FCOIs are also self-regulated by the medical profession, but attempts at creating a centralised register for all clinical staff have been inconclusive [109, 110]. The ABPI maintains close contact with government and professional organisations [93], which support industry self-regulation of payment disclosure [64, 111], having issued joint guidelines for HCOs with the ABPI [112]. Finally, freedom of information legislation is increasingly important in gaining insights into FCOIs in the NHS [108].

## Methods

We compared the transparency of disclosure *rules*, *practices*, and *data* [55].

We examined disclosure *rules* which the ABPI and JPMA described in codes, guidelines, and press releases. We identified these documents on trade group websites and by googling “Association of the British Pharmaceutical Industry”, “ABPI”, “Japan Pharmaceutical Manufacturers Association” (日本製薬工業協会 or Nihon Seiyaku Kogyo Kyokai), “JPMA”, together with the terms “transparency” (透明性 or Tomeisei), “disclosure” (公開 or Kokai), “payments” (支払い or Shiharai), and “transfers of value”. We also consulted the 2019 publication “Pharmaceutical Association Code of Practice”, comprising the JPMA Code of Practice, the Transparency Guidelines, and a glossary of relevant regulations.

We considered the JPMA Transparency Guidelines from 2018, both the English [61] and Japanese versions [113] (cited in-text as JTG2018), and the JPMA Code from October 2019, both the English [62] and Japanese [114] versions (cited in-text as JC2019). The Guidelines were further revised in 2022, without, however, introducing changes to payment disclosure. On the other hand, significant changes were being introduced to the ABPI Code at the time of writing and therefore we considered its 2019 version (cited in-text as AC2019) [74] and the newest revision implemented in 2021 (cited in-text as AC2021) [59]. We note any differences between the two, but if no change occurred, we only mention the 2019 version.

Drawing on earlier comparative research [24, 46] and single-case studies of ABPI and JPMA disclosures [30, 31, 38], we compared the disclosure *rules* in relation to the aim of disclosure, payment recipients, and payments either included or exempted from disclosure.

We examined disclosure *practices* suggested by to-date analyses of self-regulation [55, 115–117]. First, the availability of disclosures, understood as the share of companies publishing disclosures within companies committing to disclosure rules. Second, accessibility concerned where disclosures were published online. Third, we considered the electronic format of documents with drug company disclosures. Fourth, by evidence of underreporting we meant acknowledged cases of payments which should have been reported but were not. Fifth, evidence of misreporting involved attribution of payments to incorrect recipients.

We analysed disclosure availability, accessibility, and format in 2018 as this was the latest year for which we had collected the Japanese disclosures. In so doing, in the UK, we considered (1) Disclosure UK, the ABPI’s centralised payments database; (2) company “methodological notes” which describe their approaches to disclosure and are published alongside Disclosure UK [30, 31]; and (3) any relevant disclosure guides that we could find on the ABPI website [118]. In Japan, we evaluated disclosure availability, accessibility and format using payment disclosures published on individual company websites [38, 42, 43]. No changes to disclosure availability, accessibility and format have been made by either the ABPI or the JPMA since 2018.

We investigated the evidence of underreporting and misreporting by searching the PMCPA’s open archive for any cases involving breaches of clause 24 of the ABPI Code (“Transfers of Value to Health Professionals and Healthcare Organisations”); we considered the entire period since the current disclosure regulations were introduced in the UK (2015). There was no systematic evidence documenting potential underreporting and misreporting by JPMA members. Contrastingly, because JTG2018 are not mandatory (and involve no penalties for breaches), no platform existed for reporting potential cases of payment misreporting or underreporting.

In examining disclosure *data*, we summarised company disclosures using the original ABPI and JPMA payment categories, subsequently aggregating them into UK-only, Japan-only, and shared. We converted payment values from sterling and yen to US dollar using the average yearly exchange rates published by the UK Office of National Statistics [119] and the Bank of Japan [120] (1 USD = 0.75 pound sterling and 110.4 yen). The 2021 revision of the ABPI Code broadened the scope of one payment category (“joint working” replaced with “collaborative working”) and further specified the meaning of another (“fees for service and consultancy” replaced with “contracted services”) [59]. These changes did not alter the nature or structure of the disclosure data significantly, allowing for the same approach to data analysis to be applied in future research. The JPMA have made no changes to the reporting of payment categories since 2018. In practice, some companies delayed the reporting of R&D payments using the updated categories which the JPMA introduced voluntarily to JTG2018 to reflect the new Clinical Trials Act. Nevertheless, this delay did not affect the overall reported R&D volume of payments.

Two researchers collected and analysed the data separately for each country, with interpretations agreed by the entire team.

## Results

**Fig. 1:**
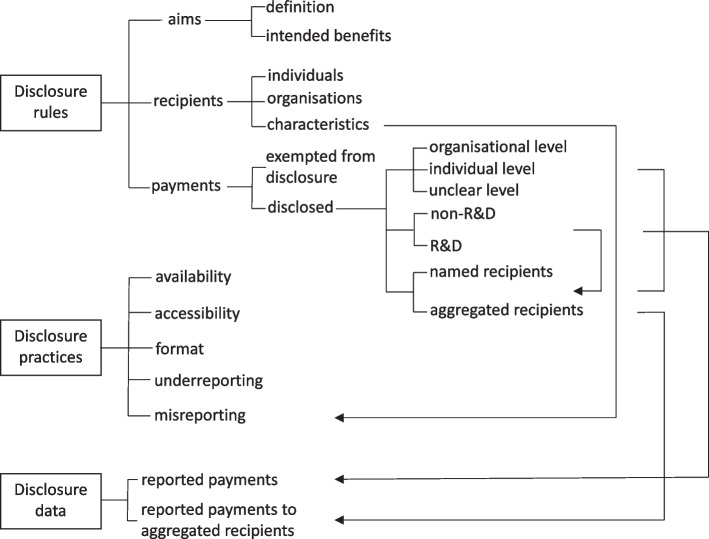
Relationships between disclosure rules, practices, and data in pharmaceutical industry self-regulation in the UK and Japan

Disclosure *rules*, *practices*, and *data* formed three distinct dimensions of transparency of self-regulated payment disclosure in the UK and Japan. Figure 1 summarises their constituent parts and relationships.

### Disclosure rules

#### Aim of disclosure

The ABPI (AC2019, Introduction) and JPMA (JTG2018, Chapter 1 – Purpose) identified transparency as the primary aim of disclosure, without, however, defining it.

According to the ABPI, transparency, or “openness”, was achieved by making payments publicly available [111, 121], and, as such, was instrumental in “building and maintaining confidence” in the industry- healthcare sector collaborations (AC2019, Introduction). Its additional benefits included demonstrating ethical behaviour, managing FCOIs in the NHS, and increasing public understanding of industry-healthcare sector collaborations [111, 121]. Likewise, the JPMA viewed transparency as key for addressing FCOIs and demonstrating commitment to ethical standards and scientific progress (JTG2018, Introduction).

#### Payment recipients

##### Recipient categories

The ABPI and JPMA distinguished individual- and organisational-level recipients. Both trade groups defined organisational-level recipients similarly, using overlapping examples (Table 1). At the individual level, however, the JPMA (JTG2018, Chapter 3 – Disclosure Recipients) defined HCPs more broadly, referring to medical personnel, while the ABPI Code mentioned professions and roles in decision-making involving medicines (AC2019, Clause 1.4). Nevertheless, in 2018 at least some companies in the UK reported payments to professionals providing treatment or interventions without prescription medicines, such as occupational health specialists, speech and language therapists, and social workers. The JPMA mentioned an additional HCP category associated with “medical operations”, corresponding with ABPI’s non-HCP “other relevant decisions makers” (ORMDs), which companies reporting their payments in Disclosure UK in 2018 interpreted as NHS administrators, managers, and board members (AC2019, Clause 1.5).

**Table 1:** Payment recipients subject to disclosure under the JPMA Transparency Guidelines and the ABPI Code

Comparative recipient categories		JPMA Transparency Guidelines (2018)	ABPI Code (2019 and 2021)
**Level of aggregation**	**Recipient categories**		
Individual	Healthcare professionals	“Medical personnel (physicians, dentists, pharmacists, public health nurses, nurses, and other persons involved in medical and nursing care)” (JTG2018, Chapter 3 – Disclosure Recipients, Medical personnel, etc.)	Anyone “who in the course of their professional activities may administer, purchase, recommend or supply a medicine” (AC2019, Clause 1.4)
	Other healthcare personnel	“Persons involved in medical operations (officers and employees of medical institutions other than medical personnel, and other persons involved in the selection or purchase of medicines)” (JTG2018, Chapter 3 – Disclosure Recipients, Medical personnel, etc.)	Other Relevant Decision Makers – “particularly includes those with an NHS [National Health Service] role who “who could influence in any way the administration, consumption, prescription, purchase, recommendation, sale, supply or use of any medicine but who are not health professionals” (AC2019, Clause 1.5)
	Non-healthcare personnel	“Life science researchers in medicine, pharmacy, science and engineering” (JTG2018, Chapter 3 – Disclosure Recipients, Medical personnel, etc.)	“Individual members of the public, including patients, individual patients not representing a patient organisation and journalists” (AC2021, Clause 4.5)
Organisational	Healthcare organisations	“Medical institutions, research institutions or departments, medical organisations, and foundations.” (JTG2018, Chapter 3 – Disclosure Recipients, (1) medical institution (2) research institution (3) Medical Organizations (4) Foundation, etc.)	“Institutions, organisations or associations that are comprised of health professionals and/or that provide healthcare or conduct research” (AC2019, Clause 19.2), such as a “hospital, clinic, foundation, university or other teaching institution or learned society” (AC2019, Clause 1.9)

If the recipient categories are the same in the 2019 and 2021 versions of the ABPI Code only the 2019 version is cited. For new recipient categories the 2021 version is cited

Two other individual-level categories did not overlap. From 2023, the ABPI provisions will extend to patients and journalists as members of the public (AC2021, Introduction). Conversely, the JPMA requires the disclosure of payments to life science researchers (JTG2018, Chapter 3 – Disclosure Recipients). While not explicitly mentioned in the ABPI Code, payments reported in Disclosure UK in 2018 suggests that at least some companies voluntarily interpreted scientists, such as biochemists and microbiologists, as HCPs or ORDMs.

##### Recipient characteristics

The ABPI’s “disclosure template” (AC2019, Clause 24.1) required the reporting of recipients’ address information and “institution name” and, optionally, “location”. Some companies reporting payments in 2018 interpreted “location” as an organisational subunit (e.g., a department within a university). The ABPI Code did not explain the difference between “names” and “locations”, potentially leading to confusion in identifying recipients [31], which could not be addressed by optional – and not published – identification numbers [122]. Further optional characteristics were HCP “speciality” and “role”, but in the absence of a shared list of categories and their descriptors companies used them inconsistently, thereby hindering or preventing reliable aggregation [24, 31]. For example, in the 2018 Disclosure UK database anaesthesiology was referred to variously as “anaesth”, “anaesthesia”, “anesthesiology” [sic], "anaesthiesia" [sic], and “anaesthetics”.

While the JPMA demanded no organisational-level characteristics (besides names), the individual-level characteristics overlapped with those required by the ABPI: affiliation and, if available, organisational sub-units, such as clinics and departments (corresponding with ABPI’s “names” and “locations”) (JTG2018, Chapter 5 – Publication details). Companies also reported recipients’ roles but without specialty and work address. Like in the UK, recipient characteristics were often missing and unstandardised in disclosures.

#### Disclosed payments

The ABPI mandated the disclosure of payments made for “promotional purposes or otherwise, in connection with the development or sale of [prescription] medicines” (AC2019, Clause 1.10, 24.1–2). In describing disclosed payments, the JPMA did not refer to promotion specifically, instead mentioning collaborations related to research or other activities undertaken by payment recipients, not necessarily in academic settings (JTG2018, Chapter 1 Background of Transparency Guidelines Enforcement; Chapter 4 – Target Payments for public disclosure).

We follow the ABPI’s and JPMA’s distinction between payments unrelated to companies’ research and development (R&D) and payments – or expenditure more broadly – related to R&D. We also compare organisational- and individual-level payments corresponding with the basic recipient categories.

#### Non-R&D payments

##### Organisational-level payments

The JPMA and ABPI mandated the disclosure of, respectively, one and four categories of non-R&D payments to HCOs (Table 2). While the JPMA category covered payments to HCOs exclusively, this was the case only for two of the four ABPI categories (donations and grants, and joint working).

**Table 2:** Non-R&D payments subject to disclosure under the JPMA Transparency Guidelines and the ABPI Code

JPMA Transparency Guidelines (2018)				ABPI Code (2019, 2021)			
**Payments**	**Recipients**	**Mode of disclosure**	**Payment description**	**Payments**	**Recipients**	**Mode of disclosure**	**Payment description**
Scholarship donations (B1)	HCOs	Named recipients	Donations to universities and research institutions for research and educational programs (Chapter 5, 3, Publication Subject, B, Academic research support expenses.)	Donations and grants	HCOs	Named recipients or aggregate recipients depending on recipient consent (starting from 2019—only named recipients)	"Medical and educational goods and services which enhance patient care, or benefit the NHS and maintain patient care" (AC2019, Clause 19.1–2). Replaced by donations (“physical items, services or benefits-in-kind)” and grants (“provision of funds”) (AC2021, Clause 1.5, 23.1)
General donations (B2)			Donations to non-profit organizations and incorporated foundations to support their operations (Chapter 5, 3, Publication Subject, B, Academic research support expenses.)				
Donations to academic and other societies (B3)			Donations to professional medical societies to support their operations (Chapter 5, 3, Publication Subject, B, Academic research support expenses.)				
Expenses related to co-sponsored conferences (B4)		Named recipients	Expenses made to host luncheon, seminars and symposiums in conferences hosted by professional medical societies (Chapter 5, 3, Publication Subject, B, Academic research support expenses)	Contributions to costs of events	HCPs and HCOs	Named recipients or aggregate recipients depending on recipient consent (only HCPs)	Organisational-level sponsorship agreements and individual-level registration fees, costs of travel, and accommodation (AC2019, Clause 22.5, 24.2). Replaced by “sponsorship” of events/meetings (organisational-level payments) and “support” of HCPs to attend events/meetings (individual-level payments), both of which can be “professional, promotional, scientific and educational” (AC2021, Clause 1.22–3, 10). Examples include “congresses, conferences, [and] symposia” (AC2021, Clause 1.7)
Expenses for lecture conferences (D1)	HCPs	Aggregate recipients	Expenses to organize conferences and symposiums for drug promotion, such as travel fees, and venue, meal, and reception costs (Chapter 5, 3, Publication Subject, D, Information provision-related expenses.)				
Lecture fees (C1)		Named recipients	Compensation to physicians for writing endeavours to other healthcare professionals, directly paid to writers (Chapter 5, 3, Publication Subject, C, Manuscript/writing fees, etc)	Fees for service and consultancy			Organisational-level "contracts between companies and institutions, organisations or associations of health professionals" (AC2019, Clause 21) and individual- or organisational-level “fees to consultants …, or to their employers on their behalf, for certain services … such as chairing and speaking at meetings, assistance with training and participation in advisory boards” (AC2019, Clause 23.2)
Manuscript writing fee/supervising fees (C2)			Compensation to physicians for advice and expertise to promote a particular medical product on the market, directly paid into the pocket of physicians (Chapter 5, 3, Publication Subject, C, Manuscript/writing fees, etc				
Consulting/commissioning fees (C3)			Compensation to physicians for speaking, training, and educational endeavours to other healthcare professionals, directly paid to lecturers (Chapter 5, 3, Publication Subject, C, Manuscript/writing fees, etc				
Other expenses related to academic research support expenses (B5)	HCOs	Named recipients	No descriptions available	No corresponding ABPI payment categories			
Other Information provision-related expenses (D4)	HCPs	Aggregate recipients					
Explanation meeting expenses (D2)			Expenses to organize study seminars for drug promotion (Chapter 5, 3, Publication Subject, D, Information provision-related expenses.)	Payments for food and drink below £75 (100$) per head are excluded from disclosure requirements. Higher payments are prohibited (AC2019, Clause 1.10, 22.1–2)			
Expenses for provision of literature and other products (D3)			Expenses for articles and documents in medicine/pharmacy, and advertising products such as pens and calendars (Chapter 5, 3, Publication Subject, D, Information provision-related expenses.)	“[I]nexpensive notebooks, pens and pencils for use at [company organised scientific] meetings” with value less than £6 per head excluding Value-Added Tax (AC2019, Clause 1.10, 18.3) are not disclosed			
Other expenses (E)	HCOs and HCPs	Aggregate recipients	Expenses for hospitality, etc. as social courtesy (Chapter 5, 3, Publication Subject, E, Other expenses.)	No corresponding ABPI payment categories			
No corresponding JPMA payment categories				Joint working (replaced by collaborative working)	HCOs	Named recipients or aggregate recipients per company depending on recipient consent (starting from 2019—only named recipients)	"Situations where, for the benefit of patients, one or more pharmaceutical companies and the NHS pool skills, experience and/or resources for the joint development and implementation of patient centred projects and share a commitment to successful delivery" (AC2019, Clause 20). Subsumed under “*collaborative working*”, covering “initiatives which either enhance patient care or are for the benefit of patients or alternatively benefit the (…) NHS and, as a minimum, maintain patient care” (AC2021, Clause 20.1, 20.4)
				Contracted services (data disclosed starting from 2023)	Members of the public including patients and journalists	Aggregate recipients per company and recipient category	Consultancy “services such as speaking at and chairing meetings, involvement in medical/ scientific studies, clinical trials or training services, writing articles and/or publications, participation at advisory board meetings, and participation in market research where such participation may involve remuneration and/or hospitality” (AC2021, Clause 24.1)

Unless stated recipient categories from the 2019 ABPI Code are the same in the 2021 version of the Code

*HCPs* Healthcare professionals, *HCOs* Healthcare organisations, *NHS* National Health Service in the UK

The JPMA Category B covered payments for subsidising recipients’ academic activities unrelated to companies’ R&D, allowing recipients to specify the purpose of funding (JTG2018, Chapter 5, 3 – Publication Subject, B Academic research support expenses). Depending on the organisational context, this category was divided into “scholarship donations” to academic institutions, such as universities or hospitals (B1); “general donations” to foundations, and non-governmental organisations, for example, providing ballpoint pens and other items and donations to a university (B2); and “donations to academic or professional societies” made to support their activities, including meetings and lectures (B3). However, if a company event, such as a luncheon or seminar, was held at a conference organised by a professional society, the respective payments would fall under “conference co-sponsoring” (B4).

We link JPMA subcategories B1-B3 to the broader category of “donations, grants and benefits in kind” from the ABPI Code (AC2019, Clause 19.1–2, 24.2), subsequently broken down into “donations” ("physical items, services or benefits in-kind”) and grants (“funds”) (AC2021, Clause 1.5, 23.1). The ABPI category was more comprehensive as it was not limited to education or academic support, also relating to “supporting healthcare” (AC2021, Clause 23.2), and was not restricted to the types of recipients listed by the JPMA. Conversely, we interpret B4 (conference co-sponsoring) as belonging to the ABPI’s broader organisational-level category of “contributions towards the costs of meetings” (AC2019, Clause 22.1, 24.2), most recently renamed as “sponsorship agreements” (AC2021, Clause 1.4, 1.22, 10.11). The ABPI category covered meetings organised by drug companies, HCOs or “other independent organisations” more broadly (AC2021, Clause 1.22). It also included costs of subsistence for participating HCPs (AC2019, Clause 22.1, AC2021, Clause 1.4, 10.1). Contrastingly, the JPMA Code (JC2019, I-2 Medical products Promotional code, Chapter 8 Provision of goods) and the Fair Competition Code (FCC, Criteria for the Operation of Article 4 of the Code, Operational standards regarding examples of restricted offerings) banned payments for travel, attendance or entertainment related to *non-promotional* (e.g. academic) meetings organised by HCPs.

Unlike the JPMA, the ABPI Code included disclosure of “fees for service and consultancy” to organisations acting on behalf of their employees (AC2019, Clause 23.2, 23.4, 24.2) and contracts with organisations (AC2019, Clause 21, 24.2). In addition, the categories of “joint working” (AC2019, Clause 24.2 and 20), later subsumed under “collaborative working” (AC2021, Clause 20), involved projects combining resources provided by donors and recipients alike and, as such, were not mentioned by the JMPA.

##### Individual-level payments

The JPMA and ABPI each mandated the disclosure of two categories of non-R&D payments to individual-level recipients (Table 2).

The JPMA “Manuscript/writing fees” (category C) included two specific subcategories – “honorariums for lectures” (C1) and “manuscript writing or supervision” (C2) – and a more general one, “consulting and commissioning” (C3), covering other forms of consultancy, such as advisory boards (JTG, Chapter 5, 3 – Publication Subject, C, manuscript/writing fees, etc.). We consider C1-C3 as matching the ABPI’s individual-level “fees for service and consultancy” (AC2019, Clause 24.2, 23.2–4) or “contracted services” (AC2021, Clause 24), which mentioned “writing articles and/or publications” and “speaking at and chairing meetings” as examples (AC2021, Clause 24.1).

The JPMA also required the disclosure of expenses related to “information provision” (category D) (JTG2018, Chapter 5, 3 – Publication Subject, D, Information provision-related expenses). These payments covered, first, expenses for promotional meetings held by companies, including support for venue, HCPs’ attendance, travel, accommodation, and post-meeting receptions with food and drink (D1). This subcategory overlapped with ABPI’s individual-level “sponsorship” (AC2019, Clause 22.5), subsequently renamed as “support” (AC2021, Clause 1.23), of attendance at meetings/events. The overlap would only be partial because the ABPI category covered not only promotional but also scientific, educational and training meetings/events (AC2019, Clause 22.1).

Second, “explanation meetings” (D2) referred to payments for food and drink provided by company sales representatives during promotional meetings in clinical settings. Under the ABPI Code, these payments would either be excluded from disclosure, if the value of food and drink was below £75 ($100) per head, or prohibited, if the value was above that figure (AC2019, Clause 1.10, 22.1–2).

A final JPMA individual-level subcategory covered costs associated with the provision of materials to assist HCPs with their scientific research (D3). There was no clear corresponding ABPI Code category. Although donations to individuals were prohibited by the ABPI (AC2019, Clause 19.1), it was conceivable that HCPs or ORDMs could claim research materials as “expenses” associated with consultancy services (AC2019, Clause 23.4).

##### Aggregate disclosure

Following the interpretation of the UK Data Protection Act and European privacy laws by companies following the ABPI Code, all HCPs and ORDMs receiving payments could only be named in drug company disclosures depending on their consent. The value of payments lacking consent to disclosure was aggregated per category (e.g. fees for service and consultancy; support for events participation). However, since late 2021 the ABPI has recommended “legitimate interest” as the preferred lawful basis for disclosure [64]: “a company asserts their transparency commitments over the data rights of the individual HCP” [123], without seeking their explicit agreement before disclosing payments. Nevertheless, companies “must allow individuals to exercise their right to raise objections” [123, 124]. The only exception to this rule were contracted services delivered by members of the public, a category introduced by the newest ABPI Code, and lacking a corresponding JPMA category (AC2021, Clause 24). Unlike payments to HCPs and ORDMs, these payments were aggregated by default (AC2021, Clause 24.6).

In Japan, only the individual recipients of “Manuscript/writing fees” (category C) were named. The JPMA informally advised its member companies to seek consent regarding the publication of their names. However, this was not an explicit rule, and data on the percentage of individuals who refused disclosure was not published. In practice, many companies accepted that HCPs could not receive payments unless they agreed for their names to be disclosed.

Conversely, payments related to “information provision” (category D) were reported as a lump sum per company. Another aggregated category – and lacking a corresponding ABPI Code category – involved expenses for social courtesies (JTG2018, Chapter. 5. E – Other expenses), including expenses for congratulations and condolences, and provision of food and beverages. This was an annual total that was aggregated and did not differentiate costs for either HCPs or HCOs.

##### R&D payments and expenditure

The JPMA and ABPI disclosure requirements covered payments for clinical trials, such as consultancy fees, which, under the JMPA Transparency guidelines would fall under “clinical trials” and “specific clinical research (AC2019, Clause 23.2; JTG, Chapter 5, 3 – Publication Subject, A, R&D expenses, etc.) (Table 3). In addition, both trade groups’ requirements encompassed non-clinical studies (AC2019, Clause 23.2; JTG, Chapter 5, 3 – Publication Subject, A, R&D expenses, etc.). In practice, JPMA members and companies following the ABPI Code disclosed payments related to both company- and investigator-initiated or sponsored studies [46].

**Table 3:** R&D payments subject to disclosure under in the JPMA Transparency Guidelines and the ABPI Code

JPMA Transparency Guidelines (2018)		ABPI Code (2019, 2021)	
**Research activities**	**Reported information**	**Research activities**	**Reported information**
Clinical trials, post-marketing clinical trials, adverse drug reaction/infection case reports, and post-marketing surveillance conducted under GCP/GVP/GPSP ordinances	Organisational-level recipients, number of contracts, value of payments (JTG2018, Chapter 5, 3, Publication Subject, A, Research and development expenses, etc)	Clinical trials defined consistent with Directive 2001/20/EC [125]	Total value of all R&D payments per company, without naming recipients or distinguishing organisational and individual recipients
Specific clinical research conducted under the Clinical Trials Act [73]	Organisational-level recipients, organisations conducting research, research Identification number recorded in the Japan Registry of Clinical Trials, the name and affiliation of the principal investigator of the research institution, the name of the contract research organization number of contracts, value of funding (JTG2018, Chapter 5, 3, Publication Subject, A, Research and development expenses, etc)		
Research carried out under the Ethical Guidelines for Medical and Health Research involving Human Subjects issued by Ministry of Health, Labour and Welfare [126]	Name of facility to which funds are provided, number of contracts, value of funding (JTG2018, Chapter 5, 3, Publication Subject, A, Research and development expenses, etc)	Prospective non-interventional studies	
Non-clinical research, for example basic research, pharmaceutical research	Name of facility receiving funding (JTG2018, Chapter 5, 3, Publication Subject, A, Research and development expenses, etc)	Non-clinical (laboratory) studies following the OECD Principles of Good Laboratory Practice [127]	
Other costs	Costs associated with meetings that are not paid to medical institutions, etc. (e.g., venue fees, food and beverage costs, and travel expenses; costs of tests, etc. not paid to medical institutions) (JTG2018, Chapter 5, 3, Publication Subject, A, Research and development expenses, etc)	No equivalent ABPI payment category	-

*GCP* Good clinical practice, *GVP* Good Vigilance Practice, *GPSP* Good Post-Marketing Surveillance Practices, *OECD* Organisation for Economic Co-operation and Development, *R&D* Research and development

Finally, the ABPI Code category of prospective non-interventional studies would fall under the broader JPMA category of medical research involving human subjects, understood as any clinical research complying with the Ministry of Health, Labour and Welfare’s Ethical Guidelines for Medical Research Involving Human Subjects, which had to be followed in the conduct of virtually all medical research, whether interventional, observational, prospective, or retrospective [128].

The ABPI expected aggregate disclosure of R&D payments as a lump sum per company (AC2019, Clause 23.2). Nevertheless, reporting practices suggested that some of the key elements of what is commonly understood to be *R&D expenditure* [46] were excluded by some companies, for example, honoraria to contract research organisations and payments to study sites [46]. On the other hand, the ABPI allowed companies to decide which costs were considered “subsidiary” to R&D activities, and therefore reported as R&D payments (AC2019, Clause 23.2), or reported as non-R&D payments, and therefore potentially on a name basis.

Contrastingly, the JPMA specified which payments were to be reported as either R&D or non-R&D (categories B – academic research support expenses- and C – lecture, writing or consulting fees), with category A (R&D) covering only research specifically contracted to and conducted by medical institutions (JTG2018, Chapter 5, 3 – Publication Subject, A, Research and development expenses, etc.). The JPMA mandated the disclosure of organisational recipient names and clinical trial numbers. The investigators names were to be disclosed, too, but not their individual fees. Importantly, not only did the JPMA mandate the disclosure of R&D payments (e.g. consultancies paid to clinical trial investigators) but *R&D expenditure* including “research funds” and “expenses” paid to medical institutions for outsourced research. Minor exceptions from disclosure included loan of equipment and damages paid to clinical trial participants.

#### Payments explicitly exempted from disclosure

Both industry trade groups listed the same or corresponding payments as excluded from disclosure.

The ABPI and JPMA excluded payments related to drug samples (AC2019, Clause 1.10; JTG2018, Chapter 4 – Target Payments for public disclosure). The ABPI also excluded market research, understood as “collection and analysis of information [on medicines]” in instances “where the company does not know the identity of the participants” (AC2019, Clause 12.2, 23.3). While the JPMA did not use the term “market research”, it would ordinarily fall under subcategories C1-C3 (JTG2018, Chapter 5, 3 – Publication Subject, C, Manuscript/writing fees, etc.). Further, under the ABPI Code companies did not need to report payments regarding “items which are to be passed on to patients and which are part of a formal patient support programme” (AC2019, Clause 18.2), “inexpensive notebooks, pens and pencils for use at those meetings” (AC2019, Clause 18.3) and subsistence below £75 ($100) per head (AC2019, Clause 22.1). These payments would be covered by JPMA’s category D (JTG2018, Chapter 5, 3 – Publication Subject, D, Information provision-related expenses).

Some exemptions were only mentioned by either trade group. While companies following the ABPI Code were not expected to disclose payments regarding over-the-counter medicines and "ordinary course purchases and sales of medicines”, these payments were excluded implicitly by the JPMA by not being mentioned in the Transparency Guidelines (AC2019, Clause 1.10).

The JPMA excluded from disclosure investigational drugs, support for membership fees, advertising fees, and capital for exhibition fees at conferences (JTG2018, Chapter 4 – Target Payments for public disclosure). Although not regulated by the ABPI, some companies reported investigational drugs as R&D payments, while others excluded them from disclosure [46].

### Disclosure practices

#### Availability

In 2018, 122 companies published their payments in Disclosure UK, the ABPI’s online platform for payment disclosure [129]. Among the disclosing companies were 63 of the 67 ABPI members (94%) and 59 non-members disclosing payments voluntarily. Four of the 67 ABPI members (6.0%) – Adaptimmune, Daval International, Sintetica, and Stallergenes Greer – did not submit disclosure reports. As they joined the ABPI towards the end of 2018, it is likely that none of the payments they might have made fell under the disclosure requirements.

In 2018, the JPMA had 72 members, all of which published payment disclosures in accordance with to the JTG2018. However, the total number of disclosing companies was 86 as it included subsidiaries and affiliates. Some of the disclosures were published late, contrary to the JTG2018. As no comprehensive disclosure platform existed, we could not tell whether other companies complied with JTG2018 voluntarily.

An unknown number of pharmaceutical companies did not subscribe to the trade groups’ disclosure codes and therefore did not disclose payments. According to the ABPI [74] and JPMA [130], disclosing companies formed a vast majority, especially of larger ones, but important exceptions included Vertex in the UK and Gilead in Japan.

#### Accessibility

The ABPI and JPMA leave gathering disclosures to companies. The ABPI collates and integrates company disclosures [131], and subsequently publishes them as Disclosure UK, a centralised database searchable by company and recipient names [132]. Starting from 2023, aggregated payments for services rendered by members of the public will be disclosed on individual company websites, that is, separately from Disclosure UK [59].

JPMA members disclose all payments in disclosure reports published on their websites. The JPMA prohibits companies from requiring registration or entering additional information to access the disclosure data (JTG2018, Chapter 6 – Notes from the perspective of ensuring transparency). However, 28 of the 86 (30.2%) companies required registration to access the 2018 data, apparently breaching the Transparency Guidelines. One possible reason is that companies had insufficient time to comply with the new requirements prohibiting registration, which entered into force in September 21 2018, in time for the data release.

To increase disclosure data accessibility, two non-governmental organisations, Tansa and the Medical Governance Research Institute, collect disclosures made by individual companies in Japan, integrating them into the Money Database [38, 106]. It comprises data from 2016 to 2019 and is searchable by company and recipient names and payment categories [133].

#### Format

Disclosure UK is available online and downloadable for further analysis as an Excel file. By contrast, in 2018, none of JPMA members published their data in a directly analysable format, instead choosing PDF (portable document format) files or tables embedded in a webpage.

#### Evidence of underreporting

The PMCPA, the ABPI’s self-regulatory oversight body, publicised three cases of payment underreporting.

The Japanese company Astellas voluntarily admitted to failing to disclose payments to UK nurses manning patient support lines for a support program and to pharmacies in relation to patient enrolment [134]. This was part of a larger case involving a lack of oversight of, and materials produced for, patient support programs, leading to §2 rulings as the PMCPA considered Astellas had brought discredit upon and reduced confidence in the industry.

Following a complaint by an ex-employee, another Japanese company, Daiichi-Sankyo*,* was also ruled in breach of §2 and publicly reprimanded in 2019 by the PMCPA for grossly under-reporting its payments to *at least* 132 HCPs sponsored to attend conferences, including 98 who attended a five-day European Society of Cardiology (ESC) congress in Germany in 2018 [135]. In addition, at least 15 and 28 HCPs were sponsored to attend ESC congresses in 2016 and 2017. The company had paid for travel, accommodation, food, and registration fees for these 132 HCPs, but none of this was disclosed – a total of around $629,000 of which $531,600 was for 2018. As even more underreporting could have occurred, the company stated it did not have complete visibility of the total payments that had not been reported.

Finally, an ex-employee revealed that Indivior, a company that has ratified the ABPI Code, did not disclose any 2017 payments, allegedly because it did not know it had agreed to disclose them since becoming an official “non-member” in 2017 [136]. The PMCPA also ruled a breach of §2.

We could not establish whether any JPMA members had been caught underreporting or not. Notably, there was one company reporting the value of zero in category A (R&D), and one in both category C (Lecture/writing/consulting fees) and category D (information provision expenses). It is impossible to ascertain whether, in fact, no payments were made in these categories or whether payments were made but not reported.

#### Evidence of misreporting

We identified three PMCPA cases involving payments attributed to incorrect individuals in the UK, all reported by the affected HCPs. All these instances were consistent with the lack unique recipient identifiers, as described above.

Merck Sharp & Dohme reported having made payments to an individual that had received none. Apparently, the company had sponsored another individual with the same name [137]. Amgen made a similar mistake, explaining that it had sponsored another individual with the same name working in the same area [138]. Importantly, Merck Sharp & Dohme made an additional incorrect payment disclosure related to the same individual as in the above case, leading to a breach of §2 ruling by the PMCPA as the company had failed to address the underlying problems [139].

Without procedures for submitting complaints or publishing corrections, establishing whether misreporting had been identified in JMPA members’ disclosures was impossible.

### Disclosure data

#### Payments to corresponding payment categories

In 2018, the 122 companies following the ABPI Code reported payments worth almost 4.5 times less than the 86 JPMA members, including subsidiaries (Table 5 in Appendix). Another key difference was the R&D shares, constituting 74.1% of the total in the UK but only 42.7% in Japan, even though the Japanese reporting also included R&D expenditure more broadly, as explained above.

Companies following the ABPI Code reported $632,985,775 (95.1%) using payment categories corresponding with the JPMA categories, including all individual-level non-R&D payments and R&D payments (Table 5 in Appendix). JMPA members reported $2,589,064,594 (86.6%) using payment categories corresponding with the ABPI categories, including $1,018,619,673.61 (39.3%) for individual-level payments and $1,570,444,920 (60.7%) for organisational-level payments.

#### Payments to aggregated recipients

Companies following the ABPI Code reported payments worth over $144.3 m to named recipients, representing 21.7% of the total, while the respective figures for JPMA members were $1.7bn and 59.9% (Table 4).

**Table 4:** Drug company R&D and non-R&D payments disclosed under the JPMA Transparency Guidelines and the ABPI Code (2018)

JPMA payment categories	Value of payments, ($)	Value of payments –merged JPMA categories ( $)	Value of payments – merged JPMA – named recipients ($)	ABPI payment categories	Total value of payments ($)	Value of payments – named recipients ($)
Research and development (A)	1,276,805,878.8	1,276,805,878.8 (100%)	1,236,732,890.2 (96.9%)	Research and development	493,470,880.7 (100%)	0.0 (0%)
Scholarship donations (B1)	167,048,419.4	217,575,581.0 (100%)	217,575,581.0 (100%)	Donations and Grants	35,118,661.8 (100%)	35,082,449.0 (99.9%)^1^
General donations (B2)	34,517,905.3					
Donations to academic and other societies (B3)	16,009,256.3					
Expenses related to co-sponsored conferences (B4)	76,063,460.5	845,194,783.3 (100%)	76,063,460.5 (10.0%)	Contributions to costs of events	55,207,855.0 (100%)	48,774,249.8 (88.3%)
Expenses for lecture conferences (D1)	769,131,322.9					
Lecture fees (C1)	211,419,118.7	248,042,904.90 (100%)	248,042,904.90 (100%)	Fees for service and consultancy	74,931,974.0 (100%)	53,459,921.3 (71.3%)
Manuscript writing fee/supervising fees (C2)	8,973,254.3					
Consulting/commissioning fees (C3)	28,934,542.5					
Other (C)^2^	161,435.2					
Other expenses related to academic research support expenses (B5)	11,203,780.9	12,621,124.4 (100%)	11,203,780.9 (88.8%)	No corresponding ABPI payment categories		
Other Information provision-related expenses (D4)	1,417,343.5					
Explanation meeting expenses (D2)	275,045,094.6	275,045,094.6 (100%)	0.0 (0%)	Excluded from or prohibited by disclosure requirements		
Expenses for provision of literature and other products (D3)	69,080,312.8	69,080,312.8 (100%)	0.0 (0%)			
Other expenses (E)	42,814,875.4	42,814,875.4 (100%)	0.0 (0%)	No corresponding ABPI payment categories		
No corresponding JPMA payment categories				Joint working	7,003,799.0 (100%)	7,003,799.0 (100%)
Total	2,988,626,001.0 (100%)	2,988,626,001.0 (100%)	1,791,064,063.3 (59.9%)	Total	665,733,170.5 (100%)	144,320,419.0 (21.7%)

^1^ Until 2019 ABPI members could consent healthcare organisations before disclosing their payments but few companies chose to do so. Consenting healthcare organisations is currently prohibited

^2 ^Originally, Category C did not have an "Other" item, but one company recorded the amount as "Other”

In the UK, the shares of payments to named recipients ranged across the payment categories – from all or nearly all (joint working and grants and donations) to none (R&D) (Table 4). Importantly, as R&D made up almost three-quarters of all reported payments, it constituted practically all aggregated payments (97.7%). The remaining aggregated payments were predominantly non-R&D individual-level payments, representing 42.7% of all non-R&D payments to individuals (Table 6 in Appendix).

In Japan, subcategory D1 (promotional meeting expenses) attracted the largest share (64.2%) of all aggregated payments. Of the 86 companies, 46 (53.5%) apparently breached the Transparency Guidelines by aggregating some payments in category A (R&D), with the aggregated amount representing 3.1% of the total payment value (Table 4). In contrast to the UK, aggregated non-R&D payments constituted 96.6% of all aggregated payments.

The values of aggregated payments per company ranged from $0 to over $48 m in the UK and from $0 to $96 m in Japan (Tables 7 and 8 in Appendix). In the UK, 71 of the 122 companies (58.2%) reported at least 50% of their payments in aggregate, but 21 companies (17.2%) reported more than 90% (Table 9 in Appendix). The company levels of aggregated payments were lower in Japan, with 20 of the 86 companies (23.3%) reporting over 50% in aggregate. The group of companies with very high levels of aggregated payments (over 90%) was not found in Japan, with only 2 (2.3%) reporting at least 75% (Table 10 in Appendix).

The UK and Japanese shares of aggregated R&D vs non-R&D payments reported by companies were highly contrasting. Only 8 of the 122 companies following the ABPI Code (6.6%) had over 50% of all their payments constituted by aggregated non-R&D payments (Table 9 in Appendix). However, for R&D payments the corresponding number of companies was 56, representing 45.9% of all companies following the ABPI Code.

In Japan, 66 of the 86 companies (76.7%) reported at least 50% of their non-R&D payments in aggregate (categories D information provision expenses – and E – Other expenses). However, for R&D payments the corresponding number of companies was 3, which constituted 3.5% of all the 86 reporting companies (category A – R&D) (Table 8 in Appendix).

In the UK, aggregated R&D payments were considerably larger than aggregated non-R&D payments, with the value of the third quartile over 11 times larger ($145,195.9 vs $1,615,243.1 – see Table 7 in Appendix). In Japan, the opposite was true; aggregated non-R&D payments were much higher than aggregated R&D payments, with the value of the third quartile almost 60 times higher ($16,872,972.9 vs $288,770.5 – see Table 8 in Appendix).

## Discussion

In its current form, the self-regulation of payment disclosure has operated in Japan since 2011 and in the UK since 2015. In Japan, disclosure research has identified widespread FCOIs among clinical trialists [72, 140, 141] and authors of clinical practice guidelines [43, 142, 143], while in the UK – across hospitals [100], commissioning [144], expert advisory [145], and policymaking bodies [32]. However, given the overall volume of payments reported annually in each country – in 2018, close to $3bn in Japan and over $0.6bn in the UK – a significant share of FCOIs affecting healthcare actors is likely to go unnoticed. Our comparative case study of three dimensions of transparency – disclosure *rules*, *practices*, and *data* – sheds light into why this may be the case and how the transparency of payment disclosures could be improved.

The ABPI and JPMA disclosure *rules* covered a broad range of payment recipients. The ABPI mandated a greater scope of non-R&D payment disclosure. On the one hand, almost all JPMA non-R&D payments were encompassed by the ABPI categories. On the other hand, the ABPI categories often lacked corresponding JPMA categories. Conversely, the JPMA required the disclosure of not only R&D payments but also R&D expenditure. It also mandated disclosure without referring to recipient consent. Shared weaknesses included some payments being exempted from named disclosure, while others – not disclosed altogether.

Companies following the ABPI Code had more transparent disclosure *practices*. There was certainty about how many disclosures were available; they were more accessible and had an analysable format. The ABPI complaints mechanism provided some insights into possible underreporting and misreporting.

However, JPMA members generated more transparent disclosure *data*, with the overall share of payments to named recipients almost three times higher, and considerably higher company-level shares compared to the UK.

### Implications for self-regulation and public regulation

We use our findings to evaluate claims regarding key advantages self-regulation as the industry’s preferred approach to governing drug promotion, stakeholder interactions and communication, and providing information on medicines [49, 104]. We also compare self-regulation with legally mandated transparency provisions identified in the US and eleven European countries [24].

Corresponding with IFPMA’s declared emphasis on increasing transparency [49, 51, 93], the JPMA and ABPI presented transparency as disclosure’s primary aim. Nevertheless, it was unexplained how transparency was to be achieved; for example, how disclosures were expected to be used, by whom, and to what purpose [20]. Limited attention given to “transparency relations” [146] is also common in European public regulation, as evidenced by a lack of granular and easily interpretable disclosures in many countries [24]. In comparison, public regulation does eliminate non-transparency resulting from the aggregation of certain payments by default [24]. Yet, the evidence from Japan (and Australia [36]) demonstrates that mandating disclosure may not require new sunshine legislation or clarifications of data privacy laws by public authorities, as it has been the case in Europe [18, 21, 24], and can be achieved within self-regulation.

In Japan, individual-level disclosure has arguably increased the industry’s accountability by revealing FCOIs underreported by KOLs [44, 99] and clinical triallists [72, 147] as well as examples of poor FCOI management [142, 143]. Contrastingly, the ABPI only “encourages” companies to observe its new non-mandatory guidance on “legitimate interests”, promising to “further increase the transparency” of non-R&D payments, but without committing explicitly to full disclosure, at least in the short run [64]. Yet, the comparison with Japan demonstrates that, contrary to the European industry’s emphasis on protecting commercial secrecy [46], it is possible to disclose at least organisational-level R&D payments without jeopardising companies’ R&D investments. It also shows the need for rebalancing the UK transparency debate – by focusing on encouraging the full disclosure of a relatively small share of non-R&D payments to individual HCPs [64] it has neglected systemic, non-individualised, and non-itemised disclosure of R&D payments to researchers and research institutions with key roles in drug development and marketing [46].

Reflecting IFPMA’s work on “synchronisation” of national codes [51], important similarities in the ABPI and JPMA disclosure rules were demonstrated by overlapping disclosure goals, recipient and payment categories. Nevertheless, as each trade group often referred to unique industry-supported activities, the extent of overlap was uncertain. Therefore, although the corresponding payment categories attracted around 90% of payments reported in each country, more granular descriptions of disclosures would likely produce lower figures. Furthermore, the emphasis of disclosure requirements differed, with the ABPI prioritising disclosure of non-R&D payments, while the JPMA – R&D payments and expenditure. Overall, while detailed internationally shared standards are a key advantage of self-regulation over national legislation in the EFPIA ecosystem [10, 22, 45], our findings suggest that the same cannot be said about the current state of self-regulation globally.

The assertion that self-regulation is more comprehensive than public regulation [10, 49, 51, 93, 148] received mixed support. The JPMA and ABPI disclosure rules covered prescribers as well as professions and roles shaping prescription through policy decisions [18, 31]. In this respect, self-regulation exceeded the scope of the US Sunshine Act, covering only physicians, nurses, and teaching hospitals [24], as well as disclosure legislation in several European countries, such as Estonia [149], Hungary [150] or Latvia [151], focusing on personnel involved in medicine prescription, supply, or distribution. Further, while the JPMA covered life science researchers, only Danish disclosure legislation included disclosure of payments to select researchers, i.e., bioanalysts and pharmacoeconomists [152].

Nevertheless, self-regulation missed some key areas of concern [153]. Unlike in some countries with public regulation, the ABPI and JPMA did not cover payments related to over-the-counter medicines [21], medical devices, or veterinary products [24, 36]. Also excluded from disclosure were organisations targeted by the industry’s lobbying [32, 103, 154–156], which are sometimes covered by public regulation, such as the Ministry of Health (Portugal) [157] and media organisations (France) [158].

The scope of payments covered by the ABPI and JPMA was broader than in some European countries with public regulation, such as Greece [159] and Hungary [150], concentrating on event delivery or participation. Notably, R&D payment disclosure mandated by the JPMA was significantly closer to the most comprehensive French and US disclosure requirements than self-regulation in Europe (R&D payments always aggregated) or Australia (R&D payments not disclosed) [18, 36, 46]. Contrastingly, payments for food and beverage were covered by public regulation in France and the US [18], but excluded from disclosure in the UK below the threshold of $100 (£75) and disclosed in the aggregate in Japan. This is an important gap in disclosure as data disclosed under the US Sunshine Act demonstrates that payments for food and beverage are widespread [160–162] and associated with increased prescription of promoted drugs [163, 164]. Finally, US disclosure requirements also include ownership or investment interests (e.g. royalties, licenses) held by physicians or their immediate family members – about 12% of reported payments amounts in the USA – but these are not covered by the ABPI and JPMA [165].

There was mixed evidence for the claim that self-regulation is more detailed than public regulation [51]. The disclosure categories used by the ABPI and JPMA were broad and the reporting did not distinguish their constituent recipients or drug company activities, making them difficult to interpret [166]. However, only two countries with public regulation – the US and France – use comprehensive sets of specific payment categories [18]; others either disclose few specific payments (Latvia [151], Lithuania [167, 168]) or use broad categories such as “professional affiliation” (Denmark [169]) or a “value, good or right” (Portugal [157]), without itemising them in the disclosure databases [24]. In addition, consistent with earlier research [30, 31], we identified examples of ambiguous or unstandardised reporting, potentially preventable by more detailed disclosure guidelines.

We found problematic the claim that self-regulation often involves higher levels of compliance than public regulation [49, 93, 170]. The cases of ABPI members not disclosing payments demonstrate that companies may start or stop following self-regulation at any point, creating potential gaps and uncertainties in reporting. Further, while the proponents of self-regulation would expect effective scrutiny of companies’ conduct from HCPs [93], the fact that only two HCPs complained about payment misreporting suggests, more than anything, that relatively few recipients check their disclosure records in sufficient detail, potentially making the PMCPA complaints-based system insufficient to reveal this practice systematically. This interpretation is supported by frequent reporting inaccuracies associated with the absence of recipient identifiers and categories [24, 31]. Similarly, the cases of underreporting considered by the PMCPA indicate that only current or ex-employees – and not, for example, competitor companies – may have sufficient knowledge of relevant internal practices, therefore limiting the pool of potential complainants [48, 92, 93, 171].

Key examples of noncompliance in Japan included delayed disclosure publication, some companies creating barriers to disclosure access, and reporting R&D payments in the aggregate [44, 106, 143]. However, consistent with earlier concerns about difficulties in measuring companies’ compliance with the JPMA standards [91], revealing underreporting and misreporting was practically impossible without publicly available disclosure standards (“methodological notes”) used by individual companies and a complaints mechanism overseen by the trade group. Just like in the UK, misreporting might be caused by ambiguities in disclosure data. Also reflecting earlier research [54, 99], underreporting could result from the relatively narrow JPMA payment categories, encompassing fewer industry activities than the ABPI categories. Notably, two of the three UK cases of underreporting involved Japanese companies. The PMCPA characterised one of them, Astellas, as displaying “multiple organisational and cultural failings” and a corporate culture that prioritised “the bottom line” over compliance obligations and ethical norms [48].

Contrastingly, the only available evaluation of the US Open Payments database that we were aware of demonstrated little evidence of non-compliance with the reporting standards [172], suggesting the superiority of legally binding requirements associated with potential penalties over self-regulatory standards [21, 24, 46].

### Limitations

Our study has important limitations. As pharmaceutical industry self-regulation has not been mapped in Asia [51], we might have missed important comparators for the UK as the strongest case of self-regulation in Europe [24]. Further, because we compared only two cases, any strengths and weaknesses identified were only relative to the other case. Therefore, considering another comparator might have revealed disclosure aims, recipients or payments missing from disclosure. In addition, as the recipient and payment categories were unique, and often described only in general terms, we could only ascertain their “correspondence” but not “equivalence”. Consequently, the comparisons across the payment categories require cautious interpretation.

We analysed similarities and differences between the two cases descriptively, without explaining the underlying mechanisms. For example, we did not consider the company and trade group and cultures more broadly, which may be an important factor behind the varying disclosure practices [26, 51]. One key aspect of such cultures worth in-depth investigation concerns the mechanisms for collecting and checking the quality and accuracy of disclosure data.

Future research on varieties of self-regulation might employ mixed methods to examine the two-way relationship between self-regulation at the global, trade group, and company levels, as well as other transparency provisions and the “medical economy” more broadly [48, 66, 91]. Of particular importance would be to trace the development of the different forms of regulation over time [104].

## Conclusions and policy recommendations

Our study indicates that measuring the transparency of payment disclosure associated with self-regulation should triangulate disclosure *rules*, *practices*, and *data*, given the possible inconsistences and important relationships between these three dimensions.

Overall, our comparative analysis of payment disclosure in the UK and Japan offered limited evidence to support key claims concerning the strengths of self-regulation. Despite its important advantages, self-regulation seemed inferior to public regulation, which was consistent with findings from Europe and Australia [24, 36, 46].

Our comparison highlights the priority areas for improvement for both the ABPI and JPMA.

- Eliminating exemptions from disclosure.
- Removing aggregate disclosure by default.
- Introducing more granular disclosure of payments and recipients.
- Increasing the richness of disclosure data by expanding recipient characteristics, following the example of the Australian pharmaceutical industry trade group [173].
- Introducing additional standards to prevent underreporting (mandatory declarations that no payments were made) and misreporting (mandatory recipient identifiers).
- Creating stronger compliance monitoring of disclosure practices and data [51], including regular independent audits to complement the reactive, complaints-based systems [48, 63, 66, 153].
- Introducing public rewards and sanctions specific to disclosure standards, e.g. highlighting top performers and underperformers across different disclosure practices and data [148].

We identified several priority areas for the JPMA.

- Extending the list of non-R&D payment categories (e.g. a broader range of consultancy-related payments, following the example of the ABPI Code) to enhance the scope of disclosure.
- Mandating companies to publish their internal disclosure standards using the example of “methodological notes” introduced by the EFPIA and ABPI.
- Creating a searchable and downloadable payments database for all companies subscribing to the JPMA Transparency Guidelines.
- Making the Transparency Guidelines mandatory for JPMA members, including sanctions for non-compliance.
- Increasing the transparency of the complaints process by establishing an arms-length body investigating and publishing details of alleged breaches of standards, potentially modelled on the PMCPA.

Finally, we propose the following priorities for the ABPI.

- Making the receipt of payments conditional on agreeing for their publication, which seems to be the established practice followed by the JPMA members.
- Expanding and standardising the scope of internal disclosure standards described in company methodological notes [46, 51].

Our findings reinforce earlier calls for replacing industry self-regulation with sunshine legislation made in the UK and Japan [38, 65] and correspond with the UK government’s ongoing work on expanding or replacing Disclosure UK with legislation [174]. Nevertheless, as such, public regulation not a panacea. Any legislation should be premised on a comprehensive international evaluation of disclosure *rules*, *practices* and *data* [24, 46, 55, 175], including their unintended consequences [23, 176, 177]. One structured way of embedding the perspectives of HCPs, HCOs, patients, and members of the public into the policy proposals would involve developing a “theory of change” [178] capturing multifaceted relationships between disclosure, transparency, as well as the economic power of the industry and the state’s regulatory power [18–20]. In so doing, it is important to consider a range of potential goals of transparency, such as increasing the industry’s accountability to the public revealing potential FCOIs [21, 24, 26, 55, 106] and protecting the integrity of healthcare research or policymaking [20, 146, 179].

Given the evidence of the limited impact of sunshine legislation on reducing FCOIs or encouraging their understanding by the public [23], any new legislation should be complemented by educational campaigns enhancing the understanding and scrutiny of disclosure *rules*, *practices* and *data* [180]. Any sunshine legislation should be integrated – including via shared databases – with FCOI evaluation undertaken by HCOs such as hospitals, commissioning bodies, regulators, and professional organisations. No less important are solutions addressing the underlying problem of financial dependency of healthcare actors on drug company funding [18, 117, 181]. Some of the available options include FCOI management policies such as limiting or banning certain forms of financial relationships [18, 20, 26, 182].

## Data Availability

The documentary materials supporting the analysis of disclosure rules in the article are cited throughout the Findings. The analysis of disclosure practices and data was based on the Japanese Money Database https://db.tansajp.org/ as well as the Disclosure UK database https://search.disclosureuk.org.uk/ and the repository of drug company methodological notes managed by the Association of the British Pharmaceutical Industry: https://www.abpi.org.uk/reputation/disclosure-uk/methodological-notes-by-company-and-year/.

## Appendix

Tables 5, 6, 7, 8, 9, 10

**Table 5:** Distribution of payments across corresponding and non-corresponding JPMA and ABPI payment categories (2018)

Payment categories	Organisational-level payments ($)		Individual-level payments ($)		Unclear if organisational- or individual-level payments ($)		Total ($)
ABPI payment categories with corresponding JPMA categories	Donations and grants	35,118,661.84	Fee for Service and consultancy	49,188,377.90	Research and development	493,470,880.71	632,985,775.44 (95.1%)
	Contribution to costs of events	39,095,432.05	Contribution to costs of Events	16,112,422.94			
	Total	74,214,093.89	Total	65,300,800.84	Total	493,470,880.71	
ABPI payment categories without corresponding JPMA categories	Fee for service and consultancy	25,743,596.09					32,747,395.07 (4.9%)
	Joint working	7,003,798.98					
	Total	32,747,395.07					
Total – ABPI payment categories							665,733,170.53 (100.0%)
JPMA payment categories with corresponding ABPI categories	Scholarship donations (B1)	167,048,419.36	Lecture fees (C1)	211,419,118.67			2,589,064,593.79 (86.6%)
	General donations (B2)	34,517,905.28	Manuscript writing fee/supervising fees (C2)	8,973,254.30			
	Donations to academic and other societies (B3)	16,009,256.31	Consulting/commissioning fees (C3),	28,934,543.36			
	Expenses related to co-sponsored conferences (B4)	76,063,460.47	Others (C)	161,435.24			
	Research and development (A)	1,276,805,878.76	Expenses for lecture conferences (D1)	769,131,322.85			
	Total	1,570,444,920.18	Total	1,018,619,673.61			
JPMA payment categories without corresponding ABPI categories	Other expenses related to academic research support expenses (B5)	11,203,780.92	Explanation meeting expenses (D2)	275,045,094.56	Other expenses (E)	42,814,875.43	399,561,407.22 (13.4%)
			Expenses for provision of literature and other products (D3)	69,080,312.81			
			Other Information provision-related expenses (D4)	1,417,343.50			
	Total	11,203,780.92	Total	345,542,750.87	Total	42,814,875.43	
Total – JPMA payment categories							2,988,626,001.01 (100.0%)

**Table 6:** Payments to individual- and organisational-level recipients under the JPMA Transparency Guidelines and the ABPI Code (2018)

Comparative JPMA-ABPI recipient categories		JPMA Transparency Guidelines			ABPI Code		
**Recipients**	**Recipient categories**	**JPMA recipient categories**	**Total value of payments ($)**	**Value of payments to named recipients ($)**	**ABPI recipient categories**	**Total value of payments ($)**	**Value of payments to named recipients ($)**
Individual level	Healthcare professionals	Medical personnel	1,364,162,424.48 (100%)	249,488,350.76 (18.3%)	Healthcare professionals	65,300,800.84 (100.0%)	37,406,101.85 (57.3%)
	Other healthcare personnel	Persons involved in medical operations			Other relevant decision makers		
	Non-healthcare professions and roles	Life science researchers			No equivalent category		
Organisational level	Healthcare organisations	Healthcare organisations	1,581,648,701.10 (100%)	1,541,575,712.50 (97.5%)	Healthcare organisations	106,961,488.98 (100.0%)	106,914,317.19 (99.9%)
Unclear if individual or organisational level		No relevant JPMA recipient categories	42,814,875.43 (100%)	0 (0%)	Recipients of research and development payments-	493,470,880.71 (100.0%)	0 (0%)
Total			2,988,626,001.01 (100%),	1,791,064,063.27 (59.9%)		665,733,170.5 (100.0%)	144,320,419.0 (21.7%)

**Table 7:** Descriptive statistics for aggregated company-level payments reported by companies following the ABPI Code (2018)

Payment categories	Minimum ($)	Median (IQR) ($)	Maximum ($)
All aggregated payments			
Value	0.0	164,675.9 (10,975.9—1,674,936.9)	48,451,656.3
Share of all payments	0.0%	60.2% (19.2%—80.4%)	100.0%
Aggregated non-R&D payments			
Value	0.0	35,417.6 (1,700.9—145,195.9)	1,468,816.7
Share of all payments	0.0%	5.5% (1.1%—16.4%)	100.0%
Share of all aggregated payments	0.0%	20.1% (3.8%—100%)	100%
Aggregated non-R&D payments to HCPs			
Value	0.0	34,623.5 (1,700.9—145,195.9)	1,468,816.7
Share of payments to HCPs	0.0%	49.7% (14.7%—77.1%)	100.0%
Share of aggregated non-R&D payments	0.0%	100.0% (100.0%—100.0%)	100.0%
R&D payments			
Value	0.0	92,443.7 (0—1,615,243.1)	47,430,167.7
Share of all payments	0.0%	43.6% (0 – 73.3%)	100.0%
Share of all aggregated payments	0.0%	79.9% (0%—96.2%)	100.0%

*Aggregated payments* Payments without named recipients, *HCPs* Healthcare professionals, *HCOs* Healthcare organisations, *R&D* Research and development

**Table 8:** Descriptive statistics for aggregated company-level payments reported by JPMA member companies (2018)

	**Minimum**	**Median (IQR) ($)**	**Maximum**
All aggregated payments (category A, D, and E)			
Value	0.0	6,346,032.8 (1,215,424.8– 17,573,059.3)	96,159,420.3
Share of all payments	0.0%	38.1% (29.1% – 49.1%)	83.1%
Aggregated non-R&D payments (category D and E)			
Value	0.0	6,325,825.0 (1,001,734.5– 16,872,972.9)	96,096,014.5
Share of all payments	0%	36.5% (25.9%– 46.0%)	83.1%
Share of all aggregated payments	0%	99.9% (94.0%– 100%)	100%
Aggregated non-R&D payments to HCPs (category D)			
Value	0.0	5,949,062.3 (879,128.7– 15,754,217.5)	94,574,275.4
Share of all payments	0%	34.5% (23.8%– 44.2%)	80.9%
Share of all aggregated non-R&D payments	0%	94.9% (91.2%– 97.9%)	100%
Aggregated non-R&D payments (category E)			
Value	0.0	221,831.0 (24,419.9– 683,182.9)	3,456,984.4
Share of all payments	0.0%	1.4% (0.4% – 2.6%)	23.8%
Share of all aggregated non-R&D payments	0.0%	4.5% (2.0% – 8.6%)	100%
Aggregated R&D payments (category A)			
Value	0.0	978.8 (0.0– 288,770.5)	7,237,318.8
Share of all payments	0.0%	0.0% (0.0%– 1.5%)	41.4%
Share of all aggregated payments	0.0%	0.1% (0.0%– 5.2%)	92.9%

Category A of R&D is further subdivided into separate detailed categories of public disclosures. Depending on the company, there was sometimes a mix of categories that were published with named information and categories that were published in aggregate. This table aggregates the latter

Category D is expenses related to information provision

Category E is defined as other non-R&D expenses that cannot be classified in any other category. It is described as including costs for hospitality as a social ritual

*Aggregated payments* Payments without named recipients, *HCPs* Healthcare professionals, *HCOs* Healthcare organisations, *R&D* Research and development

**Table 9:** Aggregated payments – company-level data reported by companies following the ABPI Code (2018)

Company name recorded in Disclosure UK	Standardised international name recorded on drug company website	Value of aggregated payments, ($)	Aggregated payments—share of all payments, (%)	Value of aggregated non-R&D payments to HCOs and HCPs ($)	Aggregated non-R&D payments—share of all payments	Aggregated non-R&D payments—share of aggregated payments	Value of aggregated HCPs payments ($)	Aggregated HCPs payments – share of payments to HCPs	Aggregated HCPs payments – share of aggregated non-R&D payments to HCPs and HCOs	Value of R&D payments ($)	R&D—share of all payments	R&D—share of all undisclosed
Martindale Pharma	Martindale Pharma	10,224.80	100.00%	10,224.80	100.00%	100.00%	10,224.80	100.00%	100.00%		0.00%	0.00%
Avexis EU Ltd	Avexis EU	5,121.90	100.00%	5,121.90	100.00%	100.00%	5,121.90	100.00%	100.00%		0.00%	0.00%
Clinuvel (UK) Ltd	Clinuvel	1,367.10	100.00%		0.00%	0.00%		N/A	N/A	1,367.10	100.00%	100.00%
Biotest UK	Biotest	9,585,976.60	99.60%	28,135.10	0.30%	0.30%	28,135.10	77.00%	100.00%	9,557,841.50	99.30%	99.70%
Clovis Oncology UK Ltd	Clovis Oncology	975,763.80	99.40%	225,033.50	22.90%	23.10%	225,033.50	97.40%	100.00%	750,730	76.50%	76.90%
Bluebird Bio UK Limited	Bluebird Bio	1,046,010.10	98.30%		0.00%	0.00%		0.00%	N/A	1,046,010	98.30%	100.00%
Sunovion Pharmaceuticals Europe Ltd	Sunovion Pharmaceuticals Europe	997,565.00	97.80%	348,327.50	34.20%	34.90%	348,327.50	100.00%	100.00%	649,237	63.70%	65.10%
Allergan Ltd	Allergan	36,889,921.10	97.40%	935,807.80	2.50%	2.50%	935,807.80	70.30%	100.00%	35,954,113.30	95.00%	97.50%
Eisai Ltd	Eisai	5,838,665.00	96.10%	176,061.60	2.90%	3.00%	176,061.60	60.90%	100.00%	5,662,603.30	93.20%	97.00%
Indivior UK Ltd	Indivior	865,474.10	96.10%	74,798.90	8.30%	8.60%	74,798.90	80.20%	100.00%	790,675	87.80%	91.40%
Tesaro UK Ltd	Tesaro	916,484.40	95.70%	136,050.50	14.20%	14.80%	136,050.50	100.00%	100.00%	780,434	81.50%	85.20%
Otsuka Pharmaceuticals UK Ltd	Otsuka Pharmaceuticals	2,223,454.80	95.20%	186,453.80	8.00%	8.40%	186,453.80	80.60%	100.00%	2,037,001.00	87.20%	91.60%
AstraZeneca	AstraZeneca	64,712,032.10	94.60%	1,364,300.20	2.00%	2.10%	1,364,300.20	47.10%	100.00%	63,347,731.90	92.60%	97.90%
Alcon UK Ltd	Alcon	1,300,368.30	94.50%	498,527.60	36.20%	38.30%	498,527.60	100.00%	100.00%	801,840.70	58.30%	61.70%
Bristol-Myers Squibb Pharmaceuticals Ltd	Bristol-Myers Squibb	43,015,683.90	94.20%	682,542.40	1.50%	1.60%	682,542.40	46.70%	100.00%	42,333,141.50	92.70%	98.40%
Servier Laboratories Ltd	Servier Laboratories	9,416,038.10	94.10%	548,154.30	5.50%	5.80%	548,154.30	73.00%	100.00%	8,867,883.80	88.60%	94.20%
Celgene Ltd	Celgene	20,684,739	92.80%	107,653	0.50%	0.50%	107,653	12.90%	100.00%	20,577,086	92.40%	99.50%
Diurnal	Diurnal	2,166,212	92.70%		0.00%	0.00%		0.00%	N/A	2,166,212	92.70%	100.00%
GW Pharmaceuticals plc	GW Pharmaceuticals	1,143,354.20	91.80%	7,797.40	0.60%	0.70%	7,797.40	14.10%	100.00%	1,135,556.80	91.20%	99.30%
Akcea Therapeutics UK	Akcea Therapeutics	266,954.30	90.90%	34,389.60	11.70%	12.90%	34,389.60	56.20%	100.00%	232,565	79.20%	87.10%
Syner-med	Syner-Med	57,872.30	90.10%		0.00%	0.00%		N/A	N/A	57,872	90.10%	100.00%
Takeda UK Ltd	Takeda	12,866,306.90	88.40%	481,291.60	3.30%	3.70%	481,291.60	100.00%	100.00%	12,385,015.30	85.10%	96.30%
Octapharma Ltd	Octapharma	706,412.60	87.70%	59,525.40	7.40%	8.40%	59,525.40	72.90%	100.00%	646,887	80.30%	91.60%
Roche Products Limited	Roche	32,860,281.40	87.60%	1,302,844.20	3.50%	4.00%	1,302,844.20	43.40%	100.00%	31,557,437.10	84.20%	96.00%
Merz Pharma UK Ltd	Merz Pharma	3,809,689.40	85.70%	514,001.80	11.60%	13.50%	514,001.80	55.30%	100.00%	3,295,687.60	74.10%	86.50%
Janssen-Cilag Ltd	Janssen Pharmaceuticals	28,864,738.90	84.40%	1,707,603.50	5.00%	5.90%	1,707,603.50	55.20%	100.00%	27,157,135.40	79.40%	94.10%
Biogen Idec Ltd	Biogen	17,022,394.10	83.90%	534,532.30	2.60%	3.10%	534,532.30	72.10%	100.00%	16,487,861.80	81.30%	96.90%
Shionogi Limited	Shionogi	223,033.00	83.70%	44,076.60	16.50%	19.80%	44,076.60	100.00%	100.00%	178,956	67.20%	80.20%
Merck Sharp & Dohme Ltd	Merck Sharp & Dohme	27,875,141.40	83.30%	78,327.80	0.20%	0.30%	78,327.80	3.70%	100.00%	27,796,813.70	83.10%	99.70%
Shire Pharmaceuticals Ltd	Shire Pharmaceuticals	3,533,626.60	80.60%	595,532.80	13.60%	16.90%	595,532.80	100.00%	100.00%	2,938,093.80	67.10%	83.10%
Pharmasure Ltd	Pharmasure	188,530.60	80.30%	71,118.00	30.30%	37.70%	71,118.00	100.00%	100.00%	117,413	50.00%	62.30%
Pharma Mar SA	PharmaMar	123,647.10	80.30%	9,384.20	6.10%	7.60%	9,384.20	40.70%	100.00%	114,263	74.20%	92.40%
Amicus Therapeutics	Amicus Therapeutics	213,547.90	80.10%	105,430.20	39.50%	49.40%	105,430.20	70.30%	100.00%	108,118	40.50%	50.60%
PTC Therapeutics Limited	PTC Therapeutics	821,220.80	79.70%	19,591.00	1.90%	2.40%	19,591.00	22.10%	100.00%	801,630	77.80%	97.60%
LEO Laboratories Ltd	LEO Pharma	2,138,828.80	79.00%	429,337.00	15.90%	20.10%	429,337.00	80.80%	100.00%	1,709,491.80	63.20%	79.90%
Astellas Pharma Ltd	Astellas Pharma	2,435,505.40	78.70%	94,330.90	3.00%	3.90%	94,330.90	44.50%	100.00%	2,341,174.60	75.70%	96.10%
Bayer Plc	Bayer	31,660,582.20	78.50%	1,961,751.60	4.90%	6.20%	1,961,751.60	49.70%	100.00%	29,698,830.60	73.60%	93.80%
Mitsubishi Tanabe Pharma Europe Ltd	Mitsubishi Tanabe Pharma	619,928.00	78.30%		0.00%	0.00%		0.00%	N/A	619,928	78.30%	100.00%
Bial Pharma UK Ltd	Bial Pharma	530,965.80	78.00%	32,811.70	4.80%	6.20%	32,811.70	18.80%	100.00%	498,154	73.20%	93.80%
Alimera Sciences Limited	Alimera Sciences	167,083.00	77.80%	28,539.50	13.30%	17.10%	28,539.50	69.50%	100.00%	138,543	64.50%	82.90%
Intercept Pharma	Intercept Pharma	740,818.70	77.70%	15,623.30	1.60%	2.10%	15,623.30	47.20%	100.00%	725,195	76.10%	97.90%
Eli Lilly & Company Ltd	Eli Lilly	8,954,564.90	77.50%	1,260,978.70	10.90%	14.10%	1,260,978.70	56.10%	100.00%	7,693,586.20	66.50%	85.90%
Boehringer Ingelheim Ltd	Boehringer Ingelheim	6,504,936.90	77.20%	1,189,117.10	14.10%	18.30%	1,189,117.10	61.40%	100.00%	5,315,819.80	63.10%	81.70%
AbbVie Limited	AbbVie	15,016,637.00	75.80%	1,105,735.20	5.60%	7.40%	1,105,735.20	32.30%	100.00%	13,910,901.70	70.20%	92.60%
Santen UK Limited	Santen Pharmaceutical	2,277,818.70	75.00%	123,464.50	4.10%	5.40%	123,464.50	37.10%	100.00%	2,154,354.20	70.90%	94.60%
Jazz Pharma	Jazz Pharmaceuticals	672,669.40	74.70%	251,931.40	28.00%	37.50%	251,931.40	80.20%	100.00%	420,738	46.70%	62.50%
Alexion Pharma UK Ltd	Alexion Pharmaceuticals	819,412.00	73.40%	616,953.00	55.30%	75.30%	616,953.00	93.10%	100.00%	202,459	18.10%	24.70%
Daiichi Sankyo UK Ltd	Daiichi Sankyo	1,390,606.60	72.50%	53,465.40	2.80%	3.80%	53,465.40	14.30%	100.00%	1,337,141.30	69.70%	96.20%
Almirall Ltd	Almirall	1,134,393.40	72.40%	345,675.70	22.00%	30.50%	345,675.70	77.30%	100.00%	788,717.70	50.30%	69.50%
Merck Serono Ltd	Merck Serono	4,205,087.10	71.40%	665,812.60	11.30%	15.80%	665,812.60	53.50%	100.00%	3,539,274.60	60.10%	84.20%
BioMarin Europe Ltd	BioMarin Pharmaceutical	3,118,921.30	71.00%	93,463.50	2.10%	3.00%	46,291.70	8.60%	49.50%	3,025,457.80	68.90%	97.00%
IQVIA IES UK Limited	IQVIA	23,373.00	70.00%	23,373.00	70.00%	100.00%	23,373.00	70.00%	100.00%		0.00%	0.00%
Novo Nordisk Limited	Novo Nordisk	9,570,827.10	69.50%	1,142,592.40	8.30%	11.90%	1,142,592.40	45.30%	100.00%	8,428,234.60	61.20%	88.10%
Dr Falk Pharma UK Ltd	Dr Falk Pharma	155,851.20	69.10%	155,851.20	69.10%	100.00%	155,851.20	70.40%	100.00%		0.00%	0.00%
Ever Pharma UK Ltd	EVER Pharma	13,770.00	68.90%	13,770.00	68.90%	100.00%	13,770.00	68.90%	100.00%		0.00%	0.00%
Leadiant Biosciences Limited	Leadiant Biosciences	64,093.20	68.30%	3,189.80	3.40%	5.00%	3,189.80	21.40%	100.00%	60,903	64.90%	95.00%
ApoPharma Inc	ApoPharma	117,871.70	66.60%		0.00%	0.00%		0.00%	N/A	117,872	66.60%	100.00%
Sandoz Ltd	Sandoz	1,672,523.40	64.80%	15,926.50	0.60%	1.00%	15,926.50	5.10%	100.00%	1,656,596.90	64.10%	99.00%
Amgen Ltd	Amgen	6,676,709.70	62.00%	216,333.20	2.00%	3.20%	216,333.20	20.70%	100.00%	6,460,376.40	60.00%	96.80%
Novartis Pharmaceuticals UK Ltd	Novartis	18,771,370.10	61.90%	688,181.20	2.30%	3.70%	688,181.20	15.10%	100.00%	18,083,188.90	59.60%	96.30%
Profile Pharma Ltd	Profile Pharma	832,647.10	60.30%	31,287.10	2.30%	3.80%	31,287.10	56.50%	100.00%	801,360	58.00%	96.20%
Pfizer Ltd	Pfizer	23,184,710.00	60.20%	84,336.10	0.20%	0.40%	84,336.10	1.70%	100.00%	23,100,373.80	60.00%	99.60%
GlaxoSmithKline plc	GlaxoSmithKline	22,745,157.20	59.50%		0.00%	0.00%		0.00%	N/A	22,745,157.20	59.50%	100.00%
Gilead	Gilead	6,505,440.80	58.50%	1,212,679.50	10.90%	18.60%	1,212,679.50	70.20%	100.00%	5,292,761.30	47.60%	81.40%
Sanofi Aventis	Sanofi	8,015,216.90	57.20%	1,354,442.30	9.70%	16.90%	1,354,442.30	46.70%	100.00%	6,660,774.60	47.60%	83.10%
Recordati Pharmaceuticals Ltd	Recordati Pharmaceuticals	46,194.60	56.30%	46,194.60	56.30%	100.00%	46,194.60	57.20%	100.00%		0.00%	0.00%
Mundipharma International Limited	Mundipharma	146,645.70	54.70%	146,645.70	54.70%	100.00%	146,645.70	77.50%	100.00%		0.00%	0.00%
Bausch & Lomb UK Ltd	Bausch + Lomb	93,492.00	54.50%		0.00%	0.00%		0.00%	N/A	93,492	54.50%	100.00%
Teva UK Limited	Teva	2,342,104.30	54.10%	136,427.60	3.20%	5.80%	136,427.60	100.00%	100.00%	2,205,676.70	50.90%	94.20%
Concordia International Rx UK Ltd	Concordia International	64,728.20	51.30%	24,762.00	19.60%	38.30%	24,762.00	98.10%	100.00%	39,966	31.70%	61.70%
HRA Pharma UK & Ireland Ltd	HRA Pharma	95,128.00	50.00%		0.00%	0.00%		0.00%	N/A	95,128	50.00%	100.00%
Aguettant Ltd	Aguettant	21,565.70	47.40%	21,565.70	47.40%	100.00%	21,565.70	50.00%	100.00%		0.00%	0.00%
Actelion Pharmaceuticals UK Ltd	Actelion Pharmaceuticals	849,107.70	45.10%	535,930.70	28.40%	63.10%	535,930.70	82.90%	100.00%	313,177	16.60%	36.90%
Ipsen Developments Ltd	Ipsen	299,426.90	44.10%	110,836.70	16.30%	37.00%	110,836.70	32.60%	100.00%	188,590	27.80%	63.00%
A. Menarini Farmaceutica Internazionale S.r.l	A. Menarini Farmaceutica Internazionale	128,098.00	44.10%	94,690.70	32.60%	73.90%	94,690.70	50.30%	100.00%	33,407	11.50%	26.10%
Grunenthal Ltd	Grunenthal	262,966.20	41.60%	107,925.20	17.10%	41.00%	107,925.20	49.60%	100.00%	155,041	24.50%	59.00%
Britannia Pharmaceuticals	Britannia Pharmaceuticals	74,980.50	39.80%	74,833.00	39.70%	99.80%	74,833.00	86.00%	100.00%	147	0.10%	0.20%
Guerbet Laboratories Ltd	Guerbet Laboratories	103,659.40	39.50%	31,494.20	12.00%	30.40%	31,494.20	64.90%	100.00%	72,165	27.50%	69.60%
Alnylam Pharmaceuticals UK & Ireland	Alnylam Pharmaceuticals	426,896.40	38.60%	92,292.60	8.40%	21.60%	92,292.60	57.90%	100.00%	334,604	30.30%	78.40%
Accretio	Accretio	794.7	35.60%	794.7	35.60%	100.00%	794.7	100.00%	100.00%		0.00%	0.00%
Chugai Pharma UK	Chugai Pharmaceutical	217,303.10	35.40%	88,239.20	14.40%	40.60%	88,239.20	45.10%	100.00%	129,064	21.00%	59.40%
Dermal	Dermal Laboratories	72,110.20	34.50%	17,527.10	8.40%	24.30%	17,527.10	13.60%	100.00%	54,583	26.10%	75.70%
Gedeon Richter (UK) Ltd	Gedeon Richter	114,012.80	33.00%	114,012.80	33.00%	100.00%	114,012.80	80.20%	100.00%		0.00%	0.00%
GE Healthcare Limited	GE Healthcare	201,164.10	32.60%	152,954.20	24.80%	76.00%	152,954.20	100.00%	100.00%	48,210	7.80%	24.00%
CSL Behring	CSL Behring	222,579.20	31.50%	118,769.70	16.80%	53.40%	118,769.70	32.10%	100.00%	103,810	14.70%	46.60%
Lundbeck Ltd	Lundbeck	248,286.50	31.40%	86,423.80	10.90%	34.80%	86,423.80	16.00%	100.00%	161,863	20.50%	65.20%
Chiesi Ltd	Chiesi	2,032,217.40	28.70%	628,223.90	8.90%	30.90%	628,223.90	45.60%	100.00%	1,403,993.40	19.80%	69.10%
Besins Healthcare (UK) Ltd	Besins Healthcare	99,987.00	27.70%	99,987.00	27.70%	100.00%	99,987.00	87.00%	100.00%		0.00%	0.00%
Napp Pharmaceuticals Ltd	Napp Pharmaceuticals	774,726.20	24.40%	774,726.20	24.40%	100.00%	774,726.20	62.80%	100.00%		0.00%	0.00%
Fresenius-Kabi Ltd	Fresenius Kabi	48,412.80	23.90%	48,412.80	23.90%	100.00%	48,412.80	23.90%	100.00%		0.00%	0.00%
Norgine	Norgine	66,774.70	20.50%	33,266.10	10.20%	49.80%	33,266.10	18.40%	100.00%	33,509	10.30%	50.20%
Fresenius Medical Care (UK) Ltd	Fresenius Medical Care	20,644.90	20.20%	20,644.90	20.20%	100.00%	20,644.90	46.80%	100.00%		0.00%	0.00%
Orion Pharma (UK) Ltd	Orion Pharma	95,822.50	16.10%	5,845.40	1.00%	6.10%	5,845.40	26.10%	100.00%	89,977	15.10%	93.90%
Proveca Ltd	Proveca	7,116.20	14.10%	7,116.20	14.10%	100.00%	7,116.20	100.00%	100.00%		0.00%	0.00%
RB	Reckitt Benckiser	5,947.80	13.80%	2,436.10	5.70%	41.00%	2,436.10	6.20%	100.00%	3,511.70	8.20%	59.00%
Sobi Ltd	Sobi	92,529.20	13.40%	92,529.20	13.40%	100.00%	92,529.20	100.00%	100.00%		0.00%	0.00%
ALK-Abello Ltd	ALK-Abello	14,955.80	11.90%	3,909.10	3.10%	26.10%	3,909.10	7.70%	100.00%	11,046.70	8.80%	73.90%
Ferring Pharma	Ferring Pharmaceuticals	102,525.80	9.10%	67,058.40	6.00%	65.40%	67,058.40	9.20%	100.00%	35,467	3.20%	34.60%
Orphan Europe UK Ltd	Orphan Europe	1,714.90	6.40%	1,714.90	6.40%	100.00%	1,714.90	50.00%	100.00%		0.00%	0.00%
Consilient Health Ltd	Consilient Health	19,993.20	6.10%	19,993.20	6.10%	100.00%	19,993.20	32.70%	100.00%		0.00%	0.00%
Aegerion Pharmaceuticals Limited	Aegerion Pharmaceuticals	1,702.90	4.20%	1,702.90	4.20%	100.00%	1,702.90	100.00%	100.00%		0.00%	0.00%
Accord-UK Ltd	Accord Healthcare	8,332.00	4.00%	8,332.00	4.00%	100.00%	8,332.00	37.90%	100.00%		0.00%	0.00%
Colonis Pharma Ltd	Colonis	534.2	3.20%	534.2	3.20%	100.00%	534.2	66.70%	100.00%		0.00%	0.00%
Baxter Healthcare Ltd	Baxter Healthcare	11,800.90	2.00%	11,800.90	2.00%	100.00%	11,800.90	3.80%	100.00%		0.00%	0.00%
Lincoln Medical	Lincoln Medical	357.2	2.00%	357.2	2.00%	100.00%	357.2	50.00%	100.00%		0.00%	0.00%
Alliance Pharmaceuticals Ltd	Alliance Pharma	2,285.20	1.70%	2,285.20	1.70%	100.00%	2,285.20	18.80%	100.00%		0.00%	0.00%
Pierre Fabre Ltd	Pierre Fabre	1,202.00	1.40%	1,202.00	1.40%	100.00%	1,202.00	2.70%	100.00%		0.00%	0.00%
Flynn Pharma	Flynn Pharma	4,101.60	1.30%	2,231.10	0.70%	54.40%	2,231.10	1.70%	100.00%	1,870.50	0.60%	45.60%
Rosemont Pharmaceuticals Ltd	Rosemont Pharmaceuticals	601	1.20%	601	1.20%	100.00%	601	11.70%	100.00%		0.00%	0.00%
Bracco UK Ltd	Bracco	555.4	0.40%	555.4	0.40%	100.00%	555.4	0.60%	100.00%		0.00%	0.00%
Seqirus	Seqirus	492.7	0.40%	492.7	0.40%	100.00%	492.7	0.40%	100.00%		0.00%	0.00%
Bio Products Laboratory Ltd	Bio Products Laboratory		0.00%		0.00%	N/A		0.00%	N/A		0.00%	N/A
Galen Ltd	Galen		0.00%		0.00%	N/A		0.00%	N/A		0.00%	N/A
PARI Medical Ltd	PARI Medical		0.00%		0.00%	N/A		0.00%	N/A		0.00%	N/A
Shield TX UK Ltd	Shield Therapeutics		0.00%		0.00%	N/A		0.00%	N/A		0.00%	N/A
STD Pharmaceutical Products Ltd	STD Pharmaceutical Products		0.00%		0.00%	N/A		N/A	N/A		0.00%	N/A
Stirling Anglian Pharmaceuticals Ltd	Stirling Anglian Pharmaceuticals		0.00%		0.00%	N/A		N/A	N/A		0.00%	N/A
Thea Pharmaceuticals Ltd	Thea Pharmaceuticals		0.00%		0.00%	N/A		N/A	N/A		0.00%	N/A
Tillotts Pharma UK Ltd	Tillotts Pharma		0.00%		0.00%	N/A		N/A	N/A		0.00%	N/A
UCB Pharma Ltd	UCB Pharma		0.00%		0.00%	N/A		N/A	N/A		0.00%	N/A
Veriton Pharma	Veriton Pharma		0.00%		0.00%	N/A		N/A	N/A		0.00%	N/A
Vifor Pharma Group	Vifor Pharma		0.00%		0.00%	N/A		N/A	N/A		0.00%	N/A

The companies are sorted descendingly based on the overall share of aggregated payments

Actelion was purchased by Janssen in June 2017 and is now known as Janssen Pulmonary Hypertension

Akcea Therapeutics became a wholly owned subsidiary of Ionis Pharmaceuticals in 2021

Aegerion was acquired by Amryt Pharma in September 2019

Allergan was acquired by AbbVie in 2020 and is now known as Allergan Aesthetics

Avexis EU changed its name to Novartis Gene Therapy EU in 2020

Profile Pharma changed its name to Zambon in February 2022

Lincoln Medical chanegd name to Bioprojet UK in January 2021

Tesaro was acquired by GSK in 2019

Martindale Pharma became an Ethypharm Group Company in 2017

Sunovion Pharmaceuticals changed its name to CNX Therapeutics in 2021

*Aggregated payments* Payments without named recipients, *R&D* Research and development

**Table 10:** Aggregated payments – company-level data reported by companies following the JPMA Code

Company name	Standardised International Name recorded on drug company website	Value of aggregated payments (A & D & E) ($)	Aggregated payments—share of all payments (%)	Value of aggregated non-R&D payments (category D&E) ($)	Aggregated non-R&D payments (category D&E)—share of all payments (%)	Aggregated non-R&D payments (category D&E)—share of aggregated payments (%)	Value of aggregated non-R&D payments to HCPs (Category D—HCPs) ($)	Aggregated non-R&D payments to HCPs (Category D—HCPs)—share of all payments	Aggregated non-R&D payments to HCPs (Category D—HCPs)—share of aggregated non-R&D payments	Value of Aggregated payments (Category E—HCPs & HCOs)	Aggregated payments (Category E—HCPs & HCOs)—share of all payments	Aggregated payments (Category E—HCPs & HCOs)—share of aggregated payments
EA Pharma Co., Ltd.,	EA Pharma	17,413,922	83.14%	17,413,922.10	83.14%	100.00%	16,934,873.19	80.85%	97.25%	479,048.91	2.29%	2.75%
CELGENE CORPORATION,	Celgene	14,623,785	78.99%	13,238,120.06	71.51%	90.52%	9,781,135.70	52.83%	73.89%	3,456,984.36	18.67%	26.11%
Shire Japan KK,	Shire Pharmaceuticals	2,966,023	72.39%	2,790,852.82	68.11%	94.09%	2,776,982.95	67.78%	99.50%	13,869.87	0.34%	0.50%
Kracie Pharma, Ltd	Kracie Pharma	3,582,579	71.79%	3,581,678.14	71.77%	99.97%	3,262,506.69	65.38%	91.09%	319,171.45	6.40%	8.91%
Mylan EPD G.K	Mylan	6,520,184	70.40%	6,520,183.79	70.40%	100.00%	6,326,172.06	68.30%	97.02%	194,011.73	2.09%	2.98%
TOA EIYO LTD,	Toa Eiyo	3,508,745	69.79%	3,435,278.29	68.33%	97.91%	2,627,617.59	52.27%	76.49%	807,660.70	16.07%	23.51%
Taisho Toyama Pharmaceutical Co Ltd	Taisho Toyama Pharmaceutical	16,597,647	68.98%	16,597,120.42	68.97%	100.00%	15,342,534.76	63.76%	92.44%	1,254,585.66	5.21%	7.56%
Otsuka Pharmaceutical Factory, Inc	Otsuka Pharmaceutical Factory	6,884,704	66.78%	6,877,315.57	66.71%	99.89%	6,592,638.08	63.95%	95.86%	284,677.49	2.76%	4.14%
NIHON PHARMACEUTICAL CO., LTD.,	Nihon Pharmaceutical	3,759,253	64.89%	2,509,810.90	43.32%	66.76%	2,362,612.60	40.78%	94.14%	147,198.30	2.54%	5.86%
Maruishi Pharmaceutical Co., Ltd.,	Maruishi Pharmaceutical	2,343,468	63.02%	2,303,006.16	61.93%	98.27%	2,289,985.76	61.58%	99.43%	13,020.40	0.35%	0.57%
KOWA PHARMACEUTICAL COMPANY LTD	Kowa Pharmaceuticals	15,077,379	62.98%	15,077,379.35	62.98%	100.00%	13,062,422.28	54.57%	86.64%	2,014,957.07	8.42%	13.36%
TSUMURA & CO.,	Tsumura	18,435,580	61.85%	18,435,580.08	61.85%	100.00%	18,054,929.32	60.57%	97.94%	380,650.76	1.28%	2.06%
Meiji Seika Pharma Co., Ltd.,	Meiji Seika Pharma	14,380,059	60.27%	9,283,129.91	38.91%	64.56%	8,217,558.56	34.44%	88.52%	1,065,571.35	4.47%	11.48%
Kissei Pharmaceutical Co., Ltd.,	Kissei Pharmaceutical	14,024,145	60.13%	12,360,144.23	53.00%	88.13%	10,361,946.51	44.43%	83.83%	1,998,197.72	8.57%	16.17%
Biofermin Pharmaceutical Co., Ltd	Biofermin Pharmaceutical	275,192	58.48%	275,192.21	58.48%	100.00%	252,229.91	53.60%	91.66%	22,962.30	4.88%	8.34%
Pfizer Japan Inc.,	Pfizer	58,456,238	54.88%	58,088,767.18	54.53%	99.37%	57,599,771.22	54.07%	99.16%	488,995.96	0.46%	0.84%
FUJIFILM Toyama Chemical Co., Ltd	FUJIFILM Toyama Chemical	2,118,662	53.82%	1,874,576.16	47.62%	88.48%	937,288.08	23.81%	50.00%	937,288.08	23.81%	50.00%
Takeda Pharmaceutical Company Limited.,	Takeda	63,042,432	53.71%	63,042,432.07	53.71%	100.00%	62,259,597.55	53.04%	98.76%	782,834.52	0.67%	1.24%
KYORIN Pharmaceutical Co., Ltd.,	Kyorin Pharmaceutical	10,720,914	52.77%	10,628,868.04	52.32%	99.14%	10,024,752.71	49.35%	94.32%	604,115.33	2.97%	5.68%
Bristol-Myers Squibb K.K,	Bristol-Myers Squibb	29,971,754	50.18%	29,388,030.74	49.20%	98.05%	29,023,493.46	48.59%	98.76%	364,537.28	0.61%	1.24%
AYUMI Pharmaceutical Corporation,	AYUMI Pharmaceutical	2,023,069	49.73%	2,023,069.23	49.73%	100.00%	1,977,970.68	48.62%	97.77%	45,098.55	1.11%	2.23%
Santen Pharmaceutical Co., Ltd.,	Santen Pharmaceutical	10,179,708	49.29%	9,969,835.96	48.27%	97.94%	9,642,725.64	46.69%	96.72%	327,110.32	1.58%	3.28%
BEE BRAND MEDICO DENTAL.Co.,Ltd	Bee Brand Medico Dental	19,795	48.66%	19,622.78	48.24%	99.13%	18,918.67	46.51%	96.41%	704.11	1.73%	3.59%
Wakamoto Pharmaceutical Co., Ltd.,	Wakamoto Pharmaceutical	2,244,556	48.06%	312,400.00	6.69%	13.92%	296,499.89	6.35%	94.91%	15,900.11	0.34%	5.09%
Novartis Pharma K.K.,	Novartis	46,059,783	46.80%	44,474,637.68	45.19%	96.56%	43,949,275.36	44.66%	98.82%	525,362.32	0.53%	1.18%
DAIICHI SANKYO COMPANY, LIMITED.,	Daiichi Sankyo	96,159,420	46.36%	96,096,014.49	46.33%	99.93%	94,574,275.36	45.60%	98.42%	1,521,739.13	0.73%	1.58%
Eisai Co., Ltd.,	Eisai	42,690,217	45.55%	35,452,898.55	37.83%	83.05%	33,958,333.33	36.24%	95.78%	1,494,565.22	1.59%	4.22%
Torii Pharmaceutical Co., Ltd.,	Torii Pharmaceutical	7,665,201	45.31%	7,199,367.81	42.55%	93.92%	6,397,904.66	37.81%	88.87%	801,463.15	4.74%	11.13%
TEIJIN PHARMA LIMITED.,	Teijin Pharma	12,028,670	45.15%	12,028,670.28	45.15%	100.00%	11,579,067.71	43.46%	96.26%	449,602.57	1.69%	3.74%
Kyowa Hakko Kirin Company, Limited,	Kyowa Kirin	32,108,834	44.71%	32,108,834.48	44.71%	100.00%	30,100,019.44	41.92%	93.74%	2,008,815.04	2.80%	6.26%
ZERIA Pharmaceutical Co., Ltd.,	Zeria Pharmaceutical	3,308,774	44.55%	3,308,774.35	44.55%	100.00%	3,108,254.69	41.85%	93.94%	200,519.66	2.70%	6.06%
Otsuka Pharmaceutical Co., Ltd.,	Otsuka Pharmaceuticals	48,269,344	44.15%	48,269,344.24	44.15%	100.00%	45,835,694.77	41.92%	94.96%	2,433,649.47	2.23%	5.04%
MOCHIDA PHARMACEUTICAL CO., LTD.,	Mochida Pharmaceutical	11,897,575	44.02%	11,897,575.13	44.02%	100.00%	11,197,795.26	41.43%	94.12%	699,779.87	2.59%	5.88%
POLA-Pharma.,	Pola Pharma	1,184,913	43.94%	1,183,855.95	43.90%	99.91%	1,103,215.32	40.91%	93.19%	80,640.63	2.99%	6.81%
MSD K.K.,	Merck Sharp & Dohme	46,779,000	43.24%	46,779,000.07	43.24%	100.00%	46,145,657.57	42.66%	98.65%	633,342.50	0.59%	1.35%
Fuso Pharmaceutical Industries, Ltd.,	Fuso Pharmaceutical Industries	1,001,214	43.02%	1,001,213.99	43.02%	100.00%	859,742.25	36.94%	85.87%	141,471.74	6.08%	14.13%
Janssen Pharmaceutical K.K.,	Janssen Pharmaceuticals	17,626,105	42.36%	13,342,653.98	32.06%	75.70%	13,201,639.49	31.73%	98.94%	141,014.49	0.34%	1.06%
Astellas Pharma Inc.,	Astellas Pharma	45,407,609	42.29%	42,110,507.24	39.22%	92.74%	42,074,275.36	39.19%	99.91%	36,231.88	0.03%	0.09%
Alfresa Pharma Corporation	Alfresa Pharma	789,557	40.88%	789,557.18	40.88%	100.00%	760,764.52	39.39%	96.35%	28,792.66	1.49%	3.65%
Sanofi K.K.,	Sanofi	21,834,459	39.34%	21,834,459.30	39.34%	100.00%	21,785,766.29	39.25%	99.78%	48,693.01	0.09%	0.22%
Bayer Yakuhin, Ltd.,	Bayer	26,629,090	39.10%	26,629,090.02	39.10%	100.00%	25,870,326.41	37.99%	97.15%	758,763.61	1.11%	2.85%
Eli Lilly Japan K.K.,	Eli Lilly	47,449,666	38.45%	46,777,425.98	37.90%	98.58%	46,414,707.45	37.61%	99.22%	362,718.53	0.29%	0.78%
TAIHO PHARMACEUTICAL CO., LTD.,	Taiho Pharmaceutical	19,423,820	38.35%	16,964,923.77	33.50%	87.34%	15,584,041.93	30.77%	91.86%	1,380,881.84	2.73%	8.14%
Sumitomo Dainippon Pharma Co., Ltd.,	Sumitomo Dainippon Pharma	23,679,188	37.88%	20,925,766.86	33.48%	88.37%	19,537,706.79	31.26%	93.37%	1,388,060.07	2.22%	6.63%
Nippon Boehringer Ingelheim Co., Ltd.,	Boehringer Ingelheim	41,288,043	37.61%	41,288,043.48	37.61%	100.00%	41,089,311.60	37.42%	99.52%	198,731.88	0.18%	0.48%
Maruho Co., Ltd.,	Maruho	14,461,866	37.54%	14,461,865.66	37.54%	100.00%	14,040,456.29	36.45%	97.09%	421,409.37	1.09%	2.91%
TOYAMA CHEMICAL CO., LTD.,	Toyama Chemical	939,531	36.74%	66,948.24	2.62%	7.13%	63,923.69	2.50%	95.48%	3,024.55	0.12%	4.52%
Nippon Chemiphar Co., Ltd.,	Nippon Chemiphar	868,502	36.57%	868,501.90	36.57%	100.00%	649,543.15	27.35%	74.79%	218,958.75	9.22%	25.21%
ASAHI KASEI PHARMA CORPORATION,	Asahi Kasei Pharma	15,323,641	36.42%	15,323,641.30	36.42%	100.00%	14,504,673.91	34.47%	94.66%	818,967.39	1.95%	5.34%
Nippon Zoki Pharmaceutical Cp., Ltd.,	Nippon Zoki	2,623,265	36.32%	2,621,573.48	36.29%	99.94%	2,567,429.69	35.54%	97.93%	54,143.79	0.75%	2.07%
Yakult Honsha Company, Limited.,	Yakult Honsha	3,967,391	36.20%	3,958,333.33	36.12%	99.77%	3,894,927.53	35.54%	98.40%	63,405.80	0.58%	1.60%
ASKA Pharmaceutical Co., Ltd	ASKA Pharmaceutical	4,306,431	34.17%	4,306,431.16	34.17%	100.00%	4,052,943.84	32.16%	94.11%	253,487.32	2.01%	5.89%
Hisamitsu Pharmaceutical Co., Inc.,	Hisamitsu Pharmaceutical	10,572,249	33.96%	10,572,248.75	33.96%	100.00%	9,938,856.72	31.93%	94.01%	633,392.03	2.03%	5.99%
Novo Nordisk Pharma Ltd.,	Novo Nordisk	10,852,882	33.89%	10,852,882.05	33.89%	100.00%	10,741,762.68	33.55%	98.98%	111,119.37	0.35%	1.02%
FUJIFILM RI Pharma, Co, Ltd	FUJIFILM RI Pharma	1,306,961	33.37%	1,003,295.82	25.62%	76.77%	983,575.85	25.11%	98.03%	19,719.97	0.50%	1.97%
Mitsubishi Tanabe Pharma Corporation,	Mitsubishi Tanabe Pharma	36,521,739	33.17%	36,521,739.14	33.17%	100.00%	34,048,913.05	30.92%	93.23%	2,472,826.09	2.25%	6.77%
KM Biologics Co., Ltd	KM Biologics	921,668	32.45%	460,786.59	16.22%	49.99%	458,519.94	16.14%	99.51%	2,266.65	0.08%	0.49%
AbbVie GK,	AbbVie	16,981,345	32.39%	16,562,567.82	31.59%	97.53%	15,810,942.72	30.16%	95.46%	751,625.10	1.43%	4.54%
TERUMO CORPORATION,	Terumo Corporation	6,923,109	32.34%	6,917,581.52	32.31%	99.92%	6,290,380.43	29.38%	90.93%	627,201.09	2.93%	9.07%
GlaxoSmithKline K.K.,	GlaxoSmithKline	13,906,467	31.85%	13,906,467.39	31.85%	100.00%	13,751,005.43	31.49%	98.88%	155,461.96	0.36%	1.12%
Merck Serono Co., Ltd.,	Merck Serono	2,937,777	30.99%	2,937,776.53	30.99%	100.00%	2,937,776.53	30.99%	100.00%	0.00	0.00%	0.00%
EN Otsuka Pharmaceutical Co., Ltd	EN Otsuka Pharmaceutical Company	71,257	30.58%	71,257.32	30.58%	100.00%	66,079.05	28.36%	92.73%	5,178.27	2.22%	7.27%
Nippon Kayaku Co., Ltd	Nippon Kayaku	2,812,512	30.34%	2,812,512.05	30.34%	100.00%	2,587,808.83	27.92%	92.01%	224,703.22	2.42%	7.99%
Chugai Pharmaceutical Co., Ltd.,	Chugai Pharmaceutical	41,071,879	29.35%	41,071,879.46	29.35%	100.00%	39,528,055.44	28.25%	96.24%	1,543,824.02	1.10%	3.76%
Asahi Kasei Medical Co.,Ltd	Asahi Kasei Medical	468,089	29.07%	444,990.95	27.64%	95.07%	294,257.25	18.28%	66.13%	150,733.70	9.36%	33.87%
Kaken Pharmaceutical Co., Ltd.,	Kaken Pharmaceutical	6,171,882	28.28%	6,131,466.27	28.09%	99.35%	5,607,744.07	25.69%	91.46%	523,722.20	2.40%	8.54%
AstraZeneca K.K.,	AstraZeneca	22,176,141	28.23%	22,176,140.67	28.23%	100.00%	22,169,956.30	28.22%	99.97%	6,184.37	0.01%	0.03%
UCB Japan Co., Ltd.,	UCB Pharma	3,123,206	27.10%	2,975,907.14	25.82%	95.28%	2,906,786.32	25.22%	97.68%	69,120.82	0.60%	2.32%
NIPPON SHINYAKU CO., LTD.,	Nippon Shinyaku	5,801,649	26.29%	5,776,574.34	26.18%	99.57%	5,277,143.88	23.91%	91.35%	499,430.46	2.26%	8.65%
Senju Pharmaceutical Co., Ltd	Senju Pharmaceutical	5,148,426	25.33%	5,126,615.46	25.22%	99.58%	4,822,323.58	23.73%	94.06%	304,291.88	1.50%	5.94%
Shionogi & Co,. Ltd.,	Shionogi	10,643,116	23.68%	9,981,884.05	22.21%	93.79%	9,673,913.04	21.52%	96.91%	307,971.01	0.69%	3.09%
ONO PHARMACEUTICAL CO., LTD.,	Ono Pharmaceutical	36,116,877	22.99%	36,116,877.29	22.99%	100.00%	35,193,555.22	22.40%	97.44%	923,322.07	0.59%	2.56%
Biogen Japan, Ltd	Biogen	2,661,488	22.84%	2,661,487.57	22.84%	100.00%	2,578,023.89	22.12%	96.86%	83,463.68	0.72%	3.14%
Minophagen Pharmaceutical Co.,	Minophagen Pharmaceutical	117,047	22.23%	102,494.47	19.47%	87.57%	98,727.13	18.75%	96.32%	3,767.34	0.72%	3.68%
Toray Industries, Inc.,	Toray Industries	671,091	21.99%	317,852.06	10.41%	47.36%	266,172.20	8.72%	83.74%	51,679.86	1.69%	16.26%
SANWA KAGAKU KENKYUSHO CO., LTD.,	Sanwa Kagaku Kenkyusho	3,373,423	18.57%	3,373,423.43	18.57%	100.00%	3,303,505.77	18.18%	97.93%	69,917.66	0.38%	2.07%
Kyoto Pharmaceutical Industries, Ltd	Kyoto Pharmaceutical Industries	7,541	11.82%	7,540.93	11.82%	100.00%	0.00	0.00%	0.00%	7,540.93	11.82%	100.00%
Fujimoto Pharmaceutical Corporation,	Fujimoto Pharmaceutical Corporation	194,381	11.76%	194,325.65	11.75%	99.97%	175,697.58	10.63%	90.41%	18,628.07	1.13%	9.59%
Taisho Pharmaceutical Co., Ltd.,	Taisho Pharmaceutical	837,051	7.50%	77,541.10	0.69%	9.26%	69,191.95	0.62%	89.23%	8,349.15	0.07%	10.77%
The Research Foundation for Microbial Diseases of Osaka University	The Research Foundation For Microbial Diseases of Osaka University	73,024	1.36%	69,122.31	1.29%	94.66%	55,076.16	1.03%	79.68%	14,046.15	0.26%	20.32%
Teikoku Seiyaku Co., Ltd.,	Teikoku Seiyaku	34,602	0.83%	34,602.49	0.83%	100.00%	27,826.58	0.67%	80.42%	6,775.91	0.16%	19.58%
SEIKAGAKU CORPORATION,	Seikagaku Corporation	51,634	0.41%	43,530.90	0.35%	84.31%	39,760.64	0.32%	91.34%	3,770.26	0.03%	8.66%
Kowa Company. Ltd.,	Kowa Company	50,991	0.26%	49,825.33	0.25%	97.71%	41,897.97	0.21%	84.09%	7,927.36	0.04%	15.91%
Japan Tobacco Inc.,	Japan Tobacco	19,526	0.08%	19,526.21	0.08%	100.00%	0.00	0.00%	0.00%	19,526.21	0.08%	100.00%
Otsuka Holdings Co., Ltd	Otsuka Holdings	0	0.00%	0.00	0.00%	0.00%	0.00	0.00%	0.00%	0.00	0.00%	0.00%
Alcon Pharmaceuticals Ltd	Alcon	0	0.00%	0.00	0.00%	0.00%	0.00	0.00%	0.00%	0.00	0.00%	0.00%

Category A covers expenses related to Research and Development. Category D is expenses related to information provision. Category E is defined as other non-R&D expenses that cannot be classified in any other category. It is described as including costs for hospitality as a social ritual

Company name in 2018, respectively

Toray consists of the sum of the Pharmaceuticals and Medical Products Divisions

Merck Serono Co., Ltd. changed its name to Merck Biopharma Co., Ltd. in April 2019

Sumitomo Dainippon Pharma Co., Ltd. changed its name to Sumitomo Pharma Co., Ltd. in April 2022

Japan tobacco withdrew from JPMA in March 2018

KOWA PHARMACEUTICAL COMPANY LTD. was merged into KOWA in April 2019

Taisho Toyama Pharmaceutical Co Ltd. changed its name to Taisho Pharma Co., Ltd. in April 2019

FUJIFILM Toyama Chemical Co., Ltd. was formed by the merger of FUJIFILM RI Pharma, Co, Ltd and TOYAMA CHEMICAL CO., LTD, in October 2018

## Abbreviations

ABPI: Association of the British Pharmaceutical industry
EFPIA: European Federation of Pharmaceutical Manufacturers and Associations
Fair Competition Code: FCC in Japan
GCP: Good clinical practice
GVP: Good Vigilance Practice
GPSP: Good Post-Marketing Surveillance Practices
HCO: Healthcare organisation
HCP: Healthcare professional
IFPMA: International Federation of Pharmaceutical Manufacturers and Associations
JPMA: Japan Pharmaceutical Manufacturers Association
OECD: Organisation for Economic Co-operation and Development
R&D: Research and development
